# Enzymatic chemonucleolysis for lumbar disc herniation—an assessment of historical and contemporary efficacy and safety: a systematic review and meta-analysis

**DOI:** 10.1038/s41598-024-62792-8

**Published:** 2024-06-04

**Authors:** Jordy Schol, Luca Ambrosio, Shota Tamagawa, Kieran Joyce, Clara Ruiz-Fernández, Akira Nomura, Daisuke Sakai

**Affiliations:** 1 Department of Orthopaedic Surgery, Tokai University School of Medicine, Isehara, Japan; 2 Center for Musculoskeletal Innovative Research and Advancement (C-MiRA), Tokai University School of Medicine, Isehara, Japan; 3 Operative Research Unit of Orthopaedic and Trauma Surgery, Fondazione Policlinico Universitario Campus Bio-Medico, Rome, Italy; 4 Research Unit of Orthopaedic and Trauma Surgery, Department of Medicine and Surgery, Università Campus Bio-Medico Di Roma, Rome, Italy; 5 Department of Medicine for Orthopaedics and Motor Organ, Juntendo University Graduate School of Medicine, Tokyo, Japan; 6 CÚRAM, SFI Research Centre for Medical Devices, University of Galway, Galway, Ireland; 7 School of Medicine, University of Galway, Galway, Ireland

**Keywords:** Lumbar disc herniation, Low back pain, Chemonucleolysis, Condoliase, Chymopapain, Collagenase, Outcomes research, Musculoskeletal system, Surgery

## Abstract

Lumbar disc herniation (LDH) is often managed surgically. Enzymatic chemonucleolysis emerged as a non-surgical alternative. This systematic review and meta-analysis aims to assess the efficacy and safety of chemonucleolytic enzymes for LDH. The primary objective is to evaluate efficacy through “treatment success” (i.e., pain reduction) and severe adverse events (SAEs) rates. Additionally, differences in efficacy and safety trends among chemonucleolytic enzymes are explored. Following our PROSPERO registered protocol (CRD42023451546) and PRISMA guidelines, a systematic search of PubMed and Web of Science databases was conducted up to July 18, 2023. Inclusion criteria involved human LDH treatment with enzymatic chemonucleolysis reagents, assessing pain alleviation, imaging changes, and reporting on SAEs, with focus on allergic reactions. Quality assessment employed the Cochrane Source of Bias and MINORS tools. Meta-analysis utilized odds ratios (OR) with 95% confidence intervals (CI). Among 62 included studies (12,368 patients), chemonucleolysis demonstrated an 79% treatment success rate and significantly outperformed placebo controls (OR 3.35, 95% CI 2.41–4.65) and scored similar to surgical interventions (OR 0.65, 95% CI 0.20–2.10). SAEs occurred in 1.4% of cases, with slightly higher rates in chymopapain cohorts. No significant differences in “proceeding to surgery” rates were observed between chemonucleolysis and control cohorts. Limitations include dated and heterogeneous studies, emphasizing the need for higher-quality trials. Further optimization through careful patient selection and advances in therapy implementation may further enhance outcomes. The observed benefits call for wider clinical exploration and adoption. No funding was received for this review.

## Introduction

**Figure 1:**
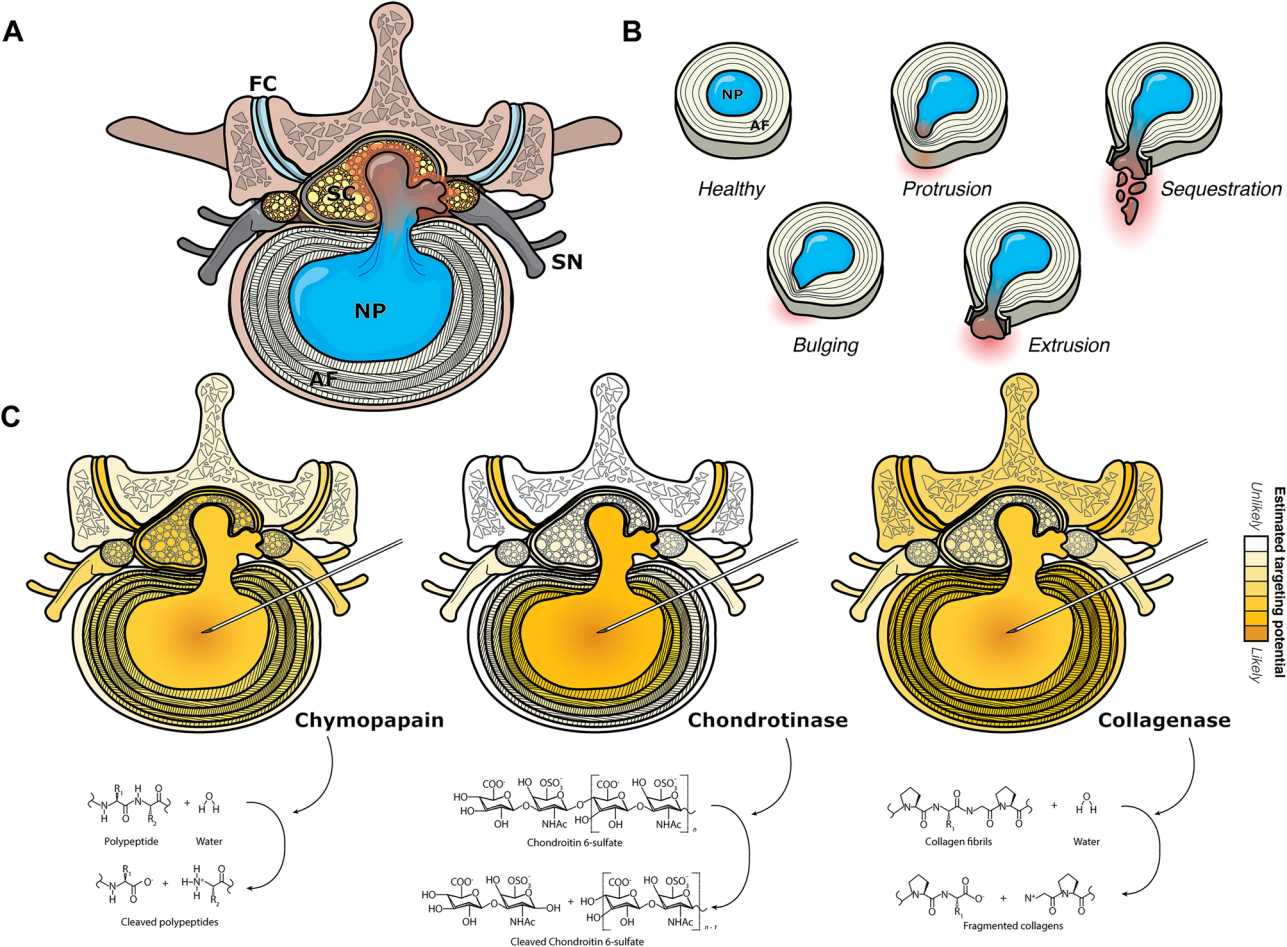
Representation of (**A**) lumbar disc herniation (LDH), (**B**) LDH types, and (**C**) representation of predicted targeted tissues for the different chemonucleolytic enzymes and their enzymatic reaction. Abbreviations: AF—annulus fibrosus, FC—facet joints, NP—nucleus pulposus, SC—spinal cord, and SN—spinal nerves.

Lumbar disc herniation (LDH) represents a pervasive spinal pathology characterized by rupture of the annulus fibrosus, resulting in the extrusion of the nucleus pulposus (NP)^1^. The consequent bulging or exposure of the NP tissue may exert physical compression or inflammation on adjacent nerve roots^2–5^, thereby inciting symptoms such as debilitating low back pain (LBP) and radiculopathy, with potential repercussions for the patient’s overall quality of life (Fig. 1A, B). The conventional management of LDH typically entails surgical interventions, often pursued following unsuccessful conservative treatments^3^. Nonetheless, surgical treatments for LDH may be accompanied by unsatisfactory outcomes in up to 15% of cases while exposing patients to not negligible risks, such as neurovascular injury and chronic post-operative LBP^6^.

In the historical context, chemonucleolysis emerged as a compelling alternative to surgical procedures, aiming to enzymatically degrade herniated disc material and assuage associated symptoms^7^. The inception of enzymatic chemonucleolysis can be attributed to chymopapain (Fig. 1C), which was introduced by Smith and Schwartz in 1964^7^ as a less invasive technique for treating LDH. This general proteolytic enzyme demonstrated promising outcomes by mitigating complications in comparison to conventional discectomy^8–10^. Despite its initial success, safety concerns surfaced, encompassing adverse reactions such as anaphylaxis and nerve root disturbances^11^. Notwithstanding, the Food and Drug Administration (FDA) retained its approval; however, the primary manufacturer terminated its production for unspecified reasons, rendering it inactive in clinical applications^12^.

Contemporary research has borne witness to a potential renaissance in chemonucleolysis, with condoliase, a chondroitin sulfate ABC endolyase (also known as chondroitinase ABC), emerging as a novel and potentially more targeted alternative. Possessing substrate-specificity for chondroitin sulfate and hyaluronic acid, condoliase selectively degrades proteoglycan-rich tissues while preserving surrounding non-proteoglycan structures^13,14^ (Fig. 1C). Approved in Japan since 2018, condoliase has undergone extensive clinical trials affirming its safety and efficacy, positioning it as a prospective advancement in LDH treatment^15,16^. Alternatively, collagenase-based therapies were also explored in the 1990s but have recently garnered some new interest as a re-explored alternative LDH-targeted chemonucleolysis therapies^17^. The evolutionary trajectory of chemonucleolysis underscores the imperative to reassess its efficacy and safety, particularly within the realm of LDH treatment.

In navigating the comparative landscape of chemonucleolysis, this systematic review and meta-analysis endeavor to evaluate the general potential and safety of chemonucleolytic treatments as an alternative, nonsurgical LDH therapy, and specifically, to discern the clinical efficacy of distinct enzymatic products. The primary objective of this systematic review is to comprehensively investigate the outcomes and trends associated with the use of chemonucleolytic enzymes in the treatment of LDH. Specifically, we aim to analyze reported data on pain alleviation, occurrence of severe adverse events (SAE), and findings from various imaging modalities following chemonucleolytic enzyme interventions. In addition, through a careful meta-analysis, our secondary objectives encompass evaluating the overall rates of SAE occurrences, treatment success rates, and the frequency of patients opting for surgery due to unsatisfactory chemonucleolytic treatment, in comparison to sham and surgery cohorts.

## Materials and methods

The study has been reported following Preferred Reporting Items for Systematic reviews and Meta-Analyses (PRISMA) guidelines^18,19^. The review protocol has been approved by the International Prospective Register of Systematic Reviews (PROSPERO) under the ID CRD42023451546^20^.

### Systematic search strategy

The initial phase involved a comprehensive systematic search of the PubMed and Web of Science databases, conducted on July 18, 2023, using a predefined syntax involving intradiscal or epidural “chemonucleolysis,” inclusive of chymopapain, condoliase, and collagenase, in conjunction with human LDH. The detailed search syntax is available in Supplemental item 1. We included eligible articles that involved (i) the injection of an enzymatic chemonucleolysis reagent, (ii) targeted treatment for LDH, and (iii) included assessments of pain, disability, imaging changes, or reported on the number of SAEs and complications following injection, with a specific focus on allergic reactions. Studies including < 10 patients, reviews, meta-analyses, case reports, letters to the editor, cadaveric studies, technical notes, preclinical studies, editorials, commentaries, and articles written in languages other than English were also excluded from the analysis.

### Study selection

**Figure 2:**
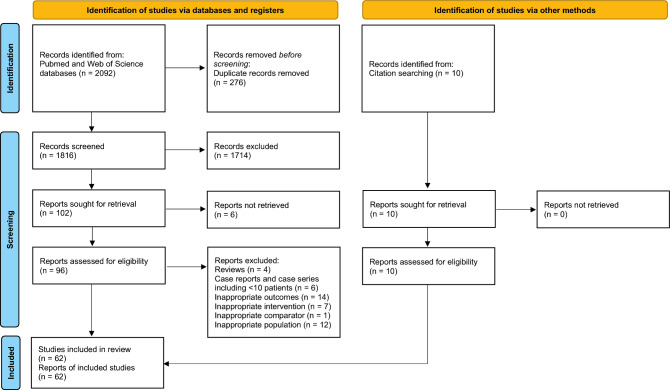
Adjusted PRISMA flowchart presenting the total number of screened papers and subsequent papers included for the systematic review and meta-analysis.

Article hits underwent automatic duplicate screening using the CADIMA software^21^. Subsequently, three researchers (JS, ST, and LA) independently screened the titles and abstracts of the identified articles against predetermined inclusion criteria. Articles suggested for inclusion by at least one of the three reviewers proceeded to the second round of full-text screening. In this stage, identical researchers conducted a comprehensive assessment to confirm adherence to the previously stated inclusion and exclusion criteria. Additionally, papers meeting the criteria were considered for inclusion in the meta-analysis if they incorporated a control group treated with (micro)discectomy, placebo or sham injection, or general conservative interventions. The article screening workflow has been reported in a PRISMA flow diagram (Fig. 2).

### Data extraction

General study characteristics extracted included: first author name, year of publication, country, funding source (if any), study design, sample size, age, sex, follow-up range (including individual timepoints, minimum, maximum, and mean, when reported), indication for chemonucleolysis, previous and concurrent treatments, technical description of the intervention (i.e., experimental product, enzyme source, injected volume and concentration, injection site, needle gauge, and number of discs injected). In comparative studies, characteristics of the control groups and respective treatments (i.e., sham injections or surgical discectomy) were recorded. Complications, success/failure rates, likelihood of undergoing further surgery, and SAEs were assessed in all included patients. Treatment success was defined as a good to excellent outcome as subjectively reported by included patients, whereas worsening or no improvement results were considered a treatment failure. An SAE was defined as a post-injection complication leading to or risking death, severely prolonging hospitalization, or causing lasting disability. Additionally, specific complications of anaphylactic shock, allergic reactions and infectious complications were separately recorded. Imaging changes (e.g., Pfirrmann classification^22^, T2-intensities at magnetic resonance imaging, disc height index, or LDH-volume/size) were also acquired. Data presented graphically were extracted using WebPlotDigitizer version 4.7 (https://automeris.io/WebPlotDigitizer, by A. Rohatgi) to approximate reported scores. Imaging outcomes were assessed at baseline and 3-, 6-, 12-, and 24-months post-transplantation, with average values, variability, and population size documented for each time point.

### Risk of bias

The Cochrane Source of Bias tool^23^ was employed to assess the quality of randomized controlled trials, while the Methodological Index for Non-Randomized Studies (MINORS) scheme^24^ was utilized to assess the risk of bias in non-randomized clinical trials. To avoid imprecision, the included papers were rated independently by one reviewer, confirmed by a second reviewer, and eventually validated by a third reviewer.

### Statistical analysis

Meta-analysis was performed using odds ratios (OR) with 95% confidence intervals (Cl) to describe dichotomous variables. The level of significance (*p*) was set at 0.05. Heterogeneity among comparisons was calculated according to the I^2^ test and was rated as “low” (I^2^ ≤ 25%), “moderate” (I^2^ = 26–74%), or “high” (I^2^ ≥ 75%). When I^2^ was ≤ 50%, a fixed-effect model was employed for analysis, whereas a random effect model was used in case of statistically significant values ≥ 50%. Pooled estimates were calculated with the Mantel–Haenszel model for treatment success rate (vs. discectomy) and rates of proceeding to surgery, while inverse variance was used for treatment success rate (vs. sham), SAE rates, and all the outcomes investigated in single-arm studies included for meta-analysis. Due to the presence of < 10 articles per each investigated outcome in comparative studies, publication bias was not evaluated. Meta-analysis was performed using the Review Manager software (v. 5.4, Cochrane Collaboration, UK) and the RStudio software (version 2023.12.1 + 402, Posit Software, MA, USA) via the metaprop package. All figures were meticulously generated using GraphPad Prism v10.0.2 (GraphPad Software LLC, USA) and Adobe Illustrator version 27.8.1 (Adobe Inc., USA).

## Results

### Literature screening

The initial literature search yielded a total of 2092 articles, resulting in 1816 articles for screening following duplicate removal (Fig. 2). Then, 1714 studies were excluded through title and abstract screening, and 6 reports could not be found, with 96 studies eventually considered for full text screening. Out of these studies, 44 were excluded (reviews, n = 4; case reports and case series including < 10 patients, n = 6; inappropriate outcomes, n = 14; inappropriate interventions, n = 7; inappropriate comparator, n = 1; inappropriate study population, n = 12). Furthermore, 10 additional papers were identified through hand citation searching and screened. Finally, 62 papers met the inclusion criteria (Table 1).

**Table 1:** Tabular overview of all included articles and their trial design sorted on the enzyme type being examined.

Article	Ref	Country	Trial type	FU_max_ (M)	Product	Funding	Patient (n)	Avg. Age (y)	Sex (M/F)	Control group (s)	Patient (n)	Avg. age (y)	Sex (M/F)
Smith (1967)	^52^	USA	Retrospective	3	Chymopapain	ns	75	ns	ns	–	–	–	–
Nordby (1972)	^53^	USA	Retrospective	12	Chymopapain	ns	100	44	64/36	Surgery	91	43	67/24
Schwetschenau (1976)	^35^	USA	RCT	11	Chymopapain	ns	31	38	19/12	Placebo	35	35	10/25
Maroon (1976)	^36^	USA	Prospective	14	Chymopapain	None	48	ns	ns	–	–	–	–
Ravichandran (1980)	^37^	UK	Prospective	9	Chymopapain	ns	26	39	19/7	–	–	–	–
Javid (1983)	^25^	USA	RCT	6	Chymopapain	ns	55	38	35/20	Placebo	53	40	28/25
Hall (1983)	^54^	Canada	Retrospective	ns	Chymopapain	ns	4282	ns	ns	–	–	–	–
Ejeskar (1983)	^26^	Sweden	RCT	12	Chymopapain	Materials provided by manufacturer	15	37	11/4	Surgery	14	42	11/3
Parkinson (1983)	^55^	Canada	Retrospective	6	Chymopapain	None	33	ns	ns	–	–	–	–
Sutton (1985a)	^56^	Canada	Retrospective	56	Chymopapain	ns	33	ns	23/10	–	–	–	–
Sutton (1985b)	^57^	Canada	Retrospective	134	Chymopapain	ns	189	39	107/82	–	–	–	–
Dabezies (1985)	^58^	USA	Retrospective	144	Chymopapain	ns	244	ns	ns	–	–	–	–
Jabaay (1985)	^38^	USA	Prospective	120	Chymopapain	ns	265	40	146/119	–	–	–	–
McDermott (1985)	^39^	USA	Prospective	6	Chymopapain	ns	1498	39	996/502	–	–	–	–
Javid (1985)	^59^	USA	Retrospective	144	Chymopapain	ns	105	ns	69/36	–	–	–	–
Lorenz (1985)	^60^	Canada	Retrospective	ns	Chymopapain	ns	55	ns	35/20	–	–	–	–
Nordby (1986)	^82^	USA	Cross–sectional	156	Chymopapain	ns	785	ns	ns	–	–	–	–
Maciunas (1986)	^40^	USA	Prospective	120	Chymopapain	ns	268	39	178/90	–	–	–	–
Hill (1986)	^61^	USA	Retrospective	2	Chymopapain	ns	335	44	ns	–	–	–	–
Dabezies (1987)	^27^	USA	RCT	6	Chymopapain	ns	78	37	57/25	Placebo	81	39	50/32
Shields (1987)	^41^	USA	Prospective	70	Chymopapain	ns	150	43	78/72	–	–	–	–
Zeiger (1987)	^62^	USA	Retrospective	46	Chymopapain	ns	45	ns	ns	Surgery	81	ns	ns
Hofstra (1989)	^63^	Netherlands	Retrospective	80	Chymopapain	None	16	43	11/5	–	–	–	–
Alexander (1989)	^64^	USA	Retrospective	35	Chymopapain	ns	51	33	46/5	Surgery	49	34	44/5
Brown (1989)	^50^	USA	Prospective	3	Chymopapain *	ns	51	38	37/14	Surgery	19	39	12/7
Boccanera (1990)	^43^	Italy	Prospective	36	Chymopapain	ns	60	34	40/20	–	–	–	–
Gogan (1991)	^28^	USA	RCT	120	Chymopapain	None	30	37	15/15	Placebo	30	37	24/6
LeBlanc (1991)	^65,101^	Canada	Retrospective	168	Chymopapain	None	50	ns	ns	–	–	–	–
Javid (1992)	^44^	USA	Prospective	48	Chymopapain	ns	106	ns	71/35	Surgery	72	41	42/30
Kato (1992)	^45,66^	Japan	Prospective	24	Chymopapain	None	26	28	22/4	–	–	–	–
Benoist (1993)	^31^	France	RCT	12	Chymopapain (Low dose)	ns	58	41	36/22	–	–	–	–
				12	Chymopapain (Standard dose)	ns	60	38	44/16	–	–	–	–
Kato (1993)	^66^	Japan	Retrospective	60	Chymopapain	ns	28	28	24/4	–	–	–	–
Leonardi (1993)	^84^	Italy	Unclear	ns	Chymopapain	ns	733	ns	ns	–	–	–	–
Benoist (1993)	^46^	France	Prospective	96	Chymopapain	ns	42	67	25/17	–	–	–	–
Louwaege (1996)	^83^	Belgium	Case series	3	Chymopapain	None	84	ns	46/38	–	–	–	–
Leivseth (1999)	^67^	Norway	Retrospective	81	Chymopapain	ns	51	ns	ns	–	–	–	–
Wittenberg (2001)	^32^	Germany	RCT	60	Chymopapain *	ns	50	36	32/18	–	–	–	–
Wardlaw (2013a)	^29,30^	UK	RCT	324	Chymopapain	ns	48	ns	ns	Surgery	52	ns	ns
Wardlaw (2013b)	^30^	UK	RCT	324	Chymopapain	ns	48	ns	27/21	Surgery	52	ns	33/19
**Chymopapain (total)**							**10,307**	**–**	**2313/1269**	**–**	**–**	**–**	**–**
Matsuyama (2018)	^33^	Japan	RCT	12	Condoliase (Low dose)	Seikagaku Corp	49	42	38/11	Placebo	47	34	31/16
				12	Condoliase (Medium dose)	Seikagaku Corp	49	38	33/16	Placebo	47	34	31/16
				12	Condoliase (High dose)	Seikagaku Corp	49	36	34/15	Placebo	47	34	31/16
Chiba (2018)	^34^	Japan	RCT	12	Condoliase	Seikagaku Corp	82	40	51/31	Placebo	81	39	48/33
Ishibashi (2020)	^68^	Japan	Retrospective	3	Condoliase	Iwai Medical Foundation	34	32	24/10	–	–	–	–
Nakajima (2020)	^69^	Japan	Retrospective	3	Condoliase	ns	42	46	29/13	–	–	–	–
Okada (2020)	^15^	Japan	Retrospective	12	Condoliase	ns	82	47	55/27	–	–	–	–
Inoue (2021)	^47^	Japan	Prospective	6	Condoliase	None	84	44	52/32	–	–	–	–
Banno (2021)	^51^	Japan	Prospective	3	Condoliase	ns	47	48	27/20	–	–	–	–
Oshita (2022)	^16^	Japan	Retrospective	3	Condoliase	None	71	ns	38/33	–	–	–	–
Takeuchi (2022)	^71,102^	Japan	Retrospective	3	Condoliase	None	101	53	80/21	–	–	–	–
Kobayashi (2022)	^72^	Japan	Retrospective	3	Condoliase	Instituional sources	127	47	88/39	–	–	–	–
Hirai (2022)	^73^	Japan	Retrospective	6	Condoliase	None	52	45	35/17	–	–	–	–
Banno (2022)	^74^	Japan	Retrospective	12	Condoliase	ns	60	45	37/23	–	–	–	–
Matsuyama (2023)	^75^	Japan	Retrospective	84	Condoliase	Seikagaku Corp	109	41	69/40	Placebo	70	40	37/33
Banno (2023)	^76^	Japan	Retrospective	33	Condoliase	ns	67	47	44/23	–	–	–	–
Kagami (2023)	^77^	Japan	Retrospective	12	Condoliase (Lateral LDH)	None	24	64	9/15	–	–	–	–
				12	Condoliase (Medial LDH)	None	133	51	61/72	–	–	–	–
Ohtonari (2023)	^78^	Japan	Retrospective	3	Condoliase	ns	47	40	31/16	–	–	–	–
Kobayashi (2023)	^79^	Japan	Retrospective	6	Condoliase	None	26	21	19/7	–	–	–	–
**Condoliase (total)**							**1,417**	**–**	**909/508**	**–**	**–**	**–**	**–**
Sussman (1981)	^48^	USA	Prospective	24	Collagenase	ns	82	37	54/28	–	–	–	–
Bromley (1982)	^80^	USA	Retrospective	11	Collagenase	ns	29	37	19/10	–	–	–	–
Bromley (1983)	^49^	USA	Prospective	30	Collagenase	ns	52	35	38/14	–	–	–	–
Brown (1985)	^42^	USA	Prospective	38	Collagenase	ns	54	36	36/18	–	–	–	–
Brown (1989)	^50^	USA	Prospective	3	Collagenase*	ns	15	35	10/5	Surgery	19	39	12/7
Zhang (2015)	^81^	China	Retrospective	3	Collagenase	ns	236	37	144/92	–	–	–	–
Wang (2021)	^17^	China	Retrospective	120	Collagenase	National Natural Science Foundation of China	126	44	84/42	–	–	–	–
Wittenberg (2001)	^32^	Germany	RCT	60	Collagenase*	ns	50	38	33/17	–	50	36	32/18
**Collagenase (total)**							**644**	**–**	**418/226**	**–**	**–**	**–**	**–**
**Chemonuclease (total)**							**12,368**	**40.7**	**3640/2003**	**–**	**–**	**–**	**–**
							**Surgery (total)**				**430**	**–**	**209/88**
							**Placebo (total)**				**397**	**–**	**228/170**
							**Combined (total)**				**827**	**38.6**	**437/258**

Cohort summaries (cumulative totals) are in bold.

*Study involving a group of collagenase and a group of chymopapain injections.

Abbreviations: Avg.—Average, Corp—Corporation, FU_max_—Maximum follow up time (indicated in months), ns—Not specified, RCT—Randomized controlled trial.

### Study characteristics

Included studies consisted of 11 RCTs^25–35^, 16 prospective studies^36–51^, 32 retrospective studies^15,17,52–81^, 1 cross-sectional study^82^, 1 case series^83^, and 1 study with an unclear design^84^ (Table 1, Fig. 2, supplemental item 2). These reports were published between 1967^52^ and 2023 from the USA^25,27,28,35,36,38–42,44,48–50,52,53,58,61,62,64,80,82^, UK^29,30,37^, Canada^54–57,60,65^, Sweden^26^, Netherlands^63^, Italy^43,84^, Japan^15,33,34,45,47,51,66,68–79^, France^31,46^, Belgium^83^, Norway^67^, Germany^32^, and China^17,81^. Within this set of articles, 39 (56.2%)^25–32,35–41,43–46,50,52–67,82–84^ involved the injection of chymopapain, 17 (26.6%)^15,33,34,47,51,68–78,85^ involved condoliase, and 8 (17.2%)^17,32,42,48–50,80,81^ involved collagenase (Table 1, supplemental item 3). Notably, the chymopapain studies were predominantly conducted in North America and Europe before 2000, whereas condoliase and collagenase studies were primarily performed in Asia, with condoliase being carried out mostly after 2015. A total of 12,368 patients with a mean age of 40.7 years were treated with chemonucleolysis. In comparative studies, the control groups consisted of 827 patients with a mean age of 38.6 years treated with either placebo, surgery, or another chemonucleolytic agent. Last follow-up ranged from a minimum of 2^61^ to 324 months^29,30^. All included patients were diagnosed single- or multilevel LDH through a combination of clinical and imaging investigations and underwent chemonucleolysis at either one or multiple disc levels (Supplemental items 2 and 4).

In chymopapain studies, the active compound was administered under the commercial names Discase (Baxter-Travenor Laboratories Inc., USA; Boots Pharmaceuticals, UK), Chymodiactin (Smith Laboratories Inc., USA; Flint Laboratories/Boots Co., USA; Boots Pharmaceuticals) when disclosed (Supplemental item 3). Injected volumes and concentrations ranged between 1.0–2.5 mL and 1000–2000 UI/mL respectively. Concentration was also reported as 4 mg/mL or 2000 pkat/mL in some studies. The final product consisted of 2000–4000 UI, 4–8 mg, or 3000–4000 pkat injected intradiscally using needles sized between 18 and 27G in most studies (Table 3). In condoliase studies, the active compound was purchased from Seigaku Corp. (Japan) under the commercial name Hernicore in the majority of studies. Injected volumes and concentrations varied between 1.0–1.2 mL and 1.0–5.0 U/mL, totaling 1.0–5.0 U eventually administered. The drug was delivered intradiscally using 21–23G needles. In collagenase studies, the drug was purchased from Advance Biofactures Corp. (USA) under the name of Nucleolysin or from Liaoning Wei Bang (China, commercial name not reported). Administered volumes and concentrations varied between 0.1–1.0 mL and 125.0–2000.0 U/mL, resulting in 5.0–600.0 U injected intradiscally with 18–22G needles.

### Risk of bias

The MINORS tool was employed to assess the quality of evidence of included nonrandomized clinical trials, with an average score of 8/16 for noncomparative studies and 16/24 for comparative studies, indicating a serious risk of bias. Likewise, according to the Cochrane Source of Bias tool, the risk of bias in included randomized controlled trials was also significant, with an average score of 18/26 (Supplemental item 5).

### Treatment success rates

**Figure 3:**
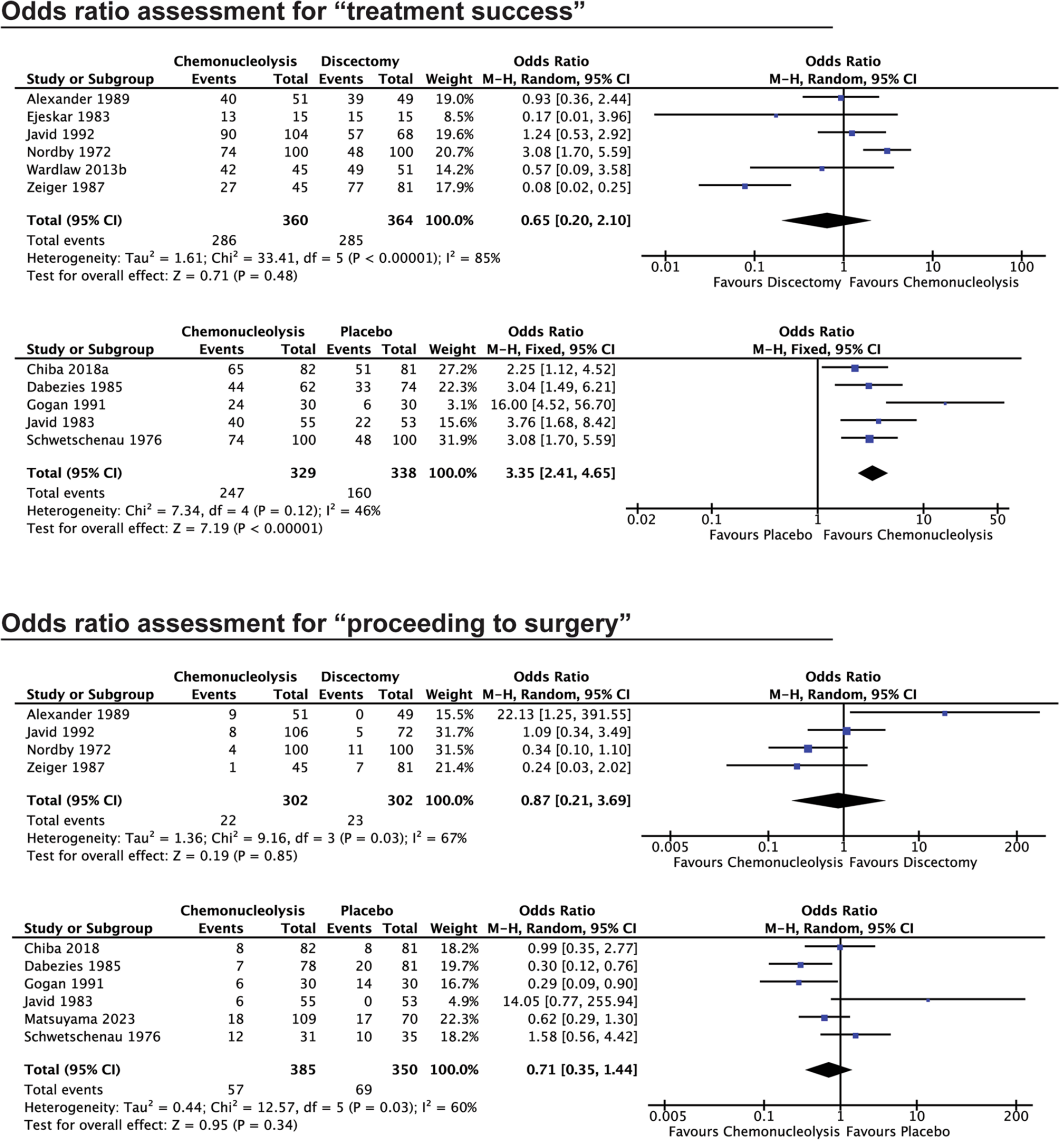
Meta-analysis forest plots depicting the assessment of (in order) the rate of treatment success of chemonucleolysis treated patients compared to surgical discectomy and placebo-treated patients, followed by plots demonstrating the rate of proceeding to surgery compared to surgical discectomy and placebo-treated patients.

Treatment success, as defined by improvement in pain from baseline, was trackable in 56/62 reports, and 59/67 experimental cohorts (Table 2, supplemental item 6). Overall, 6030/7588 chemonucleolysis procedures were reported as successful, which equates to 79% of treated patients. Similar rates were seen for chymopapain, condoliase and collagenase specifically treated patients at 79%, 78%, and 82% respectively. Eight comparative studies^26,29,42,44,50,53,62,64^ compared chemonucleolysis with surgical decompression and 5 studies^25,27,28,34,35^ compared chemonucleolysis to placebo treatments. While no statistically significant intergroup difference was found among the former (OR: 0.65, 95% CI: 0.20–2.10, *p* = 0.16; Fig. 3), chemonucleolysis showed a significantly higher treatment success rate vs. placebo injections (OR: 3.55, 95% CI: 2.41–4.65, *p* = 0.003; Fig. 3).

**Table 2:** Tabular overview of treatment success as indicated by improvement in pain outcomes. Values are given as the number of events (n) within the patient population (N) and the corresponding percentage (%).

	Article	Ref	Treatment success (n/N [%])		Treatment failure (n/N [%])		Proceeded to surgery (n/N [%])	Control	Control success (n/N [%])	Control failure (n/N [%])	Control proceeded to surgery (n/N [%])
Chymopapain	Smith (1967)	^52^	68/75 [91%]		7/75 [9%]		2/75 [3%]	–	–	–	–
	Nordby (1972)	^53^	74/100 [74%]		26/100 [26%]		4/100 [4%]	Surgery	48/100 [48%]	52/100 [52%]	11/100 [11%]
	Schwetschenau (1976)	^35^	15/31 [48%]		16/31 [52%]		12/31 [39%]	Placebo	20/35 [57%]	15/35 [43%]	10/35 [29%]
	Maroon (1976)	^36^	39/48 [81%]		9/48 [19%]		9/48 [19%]	–	–	–	–
	Ravichandran (1980)	^37^	20/26 [77%]		6/26 [23%]		5/26 [19%]	–	–	–	–
	Javid (1983)	^25^	40/55 [73%]		15/55 [27%]		6/55 [11%]	Placebo	22/53 [42%]	31/53 [58%]	0/53 [0%]
	Hall (1983)	^54^	ns		ns		ns	–	–	–	–
	Ejeskar (1983)	^26^	13/15 [87%]		2/15 [13%]		ns	Surgery	15/15 [100%]	0/15 [0%]	ns
	Parkinson (1983)	^55^	198/200 [99%]		2/200 [1%]		ns	–	–	–	–
	Sutton (1985a)	^56^	24/33 [73%]		9/33 [27%]		5/33 [15%]	–	–	–	–
	Sutton (1985b)	^57^	147/189 [78%]		42/189 [22%]		24/189 [13%]	–	–	–	–
	Dabezies (1985)	^58^	272/382 [71%]		110/382 [29%]		36/244 [15%]	–	–	–	–
	Jabaay (1985)	^38^	221/263 [84%]		42/263 [16%]		ns	–	–	–	–
	McDermott (1985)	^39^	1226/1402 [87%]		176/1402 [13%]		62/1498 [4%]	–	–	–	–
	Javid (1985)	^59^	85/105 [81%]		20/105 [19%]		17/105 [16%]	–	–	–	–
	Lorenz (1985)	^60^	44/55 [80%]		11/55 [20%]		11/55 [20%]	–	–	–	–
	Nordby (1986)	^82^	493/739 [67%]		246/739 [33%]		129/739 [17%]	–	–	–	–
	Maciunas (1986)	^40^	230/268 [86%]		38/268 [14%]		54/268 [20%]	–	–	–	–
	Hill (1986)	^61^	261/335 [78%]		74/335 [22%]		74/335 [22%]	–	–	–	–
	Dabezies (1987)	^27^	44/62 [71%]		18/62 [29%]		7/78 [9%]	Placebo	33/74 [45%]	41/74 [55%]	20/81 [25%]
	Shields (1987)	^41^	50/126 [40%]		76/126 [60%]		23/126 [18%]	–	–	–	–
	Zeiger (1987)	^62^	27/45 [60%]		18/45 [40%]		1/45 [2%]	Surgery	77/81 [95%]	4/81 [5%]	7/81 [9%]
	Hofstra (1989)	^63^	15/15 [100%]		0/15 [0%]		0/16 [0%]	–	–	–	–
	Alexander (1989)	^64^	40/51 [78%]		11/51 [22%]		9/51 [18%]	Surgery	39/49 [80%]	10/49 [20%]	0/49 [0%]
	Brown (1989)	^50^	35/51 [68%]		16/51 [32%]		6/51 [12%]	Surgery ^‹^	18/19 [95%]	1/19 [5%]	0/19 [0%]
	Boccanera (1990)	^43^	54/60 [90%]		6/60 [10%]		ns	–	–	–	–
	Gogan (1991)	^28^	24/30 [80%]		6/30 [20%]		6/30 [20%]	Placebo	9/26 [35%]	17/26 [65%]	14/30 [47%]
	LeBlanc (1991)	^65^	37/50 [74%] ^#^		13/50 [26%] ^#^		13/50 [26%]	–	–	–	–
	Javid (1992)	^44^	90/104 [87%]		14/104 [13%]		8/106 [8%]	Surgery	57/68 [84%]	11/68 [16%]	5/72 [7%]
	Kato (1992)	^45^	ns		ns		ns	–	–	–	–
	Benoist (1993) (L)	^31^	36/41 [88%]		5/41 [12%]		ns	–	–	–	–
	Benoist (1993) (M)		38/43 [88%]		5/43 [12%]		ns	–	–	–	–
	Kato (1993)	^66^	27/28 [96%]		1/28 [4%]		1/28 [4%]	–	–	–	–
	Leonardi (1993)	^84^	602/733 [82%]		131/733 [18%]		ns	–	–	–	–
	Benoist (1993)	^46^	33/42 [79%]		9/42 [21%]		2/42 [5%]	–	–	–	–
	Louwaege (1996)	^83^	61/84 [72%] ^†^		23/84 [27%] ^†^		2/84 [2%]	–	–	–	–
	Leivseth (1999)	^67^	24/50 [48%]		26/50 [52%]		ns	–	–	–	–
	Wittenberg (2001) *	^32^	36/50 [72%]		14/50 [28%]		9/50 [18%]	–	–	–	–
	Wardlaw (2013a)	^29^	ns		ns		ns	–	–	–	–
	Wardlaw (2013b)	^30^	42/45 [93%]		3/45 [7%]		9/48 [19%]	Surgery	49/51 [96%]	2/51 [4%]	ns
**Chymopapain (total)**			**4785/6031 [79%]**		**1246/6031 [21%]**		**546/4606 [11.9%]**	**–**	**–**	**–**	**–**
Condoliase	Matsuyama (2018) (L)	^33^	ns		ns		ns	–	–	–	ns
	Matsuyama (2018) (M)		ns		ns		ns	–	–	–	–
	Matsuyama (2018) (H)		ns		ns		ns	–	–	–	–
	Chiba (2018)	^34^	65/82 [79%]		17/82 [21%]		8/81 [10%]	Placebo	51/81 [63%]	30/81 [37%]	8/81 [10%]
	Ishibashi (2020)	^68^	21/34 [62%]		13/34 [38%]		6/34 [18%]	–	–	–	–
	Nakajima (2020)	^69^	32/42 [76%] ^‡^		10/42 [24%] ^‡^		ns	–	–	–	–
	Okada (2020)	^15^	70/82 [85%] ^‡^		12/82 [15%] ^‡^		4/82 [5%]	–	–	–	–
	Inoue (2021)	^47^	65/84 [77%] ^‡^		19/84 [23%] ^‡^		11/84 [13%]	–	–	–	–
	Banno (2021)	^51^	33/46 [70%] ˆ		14/47 [30%] ˆ		11/47 [23%]	–	–	–	–
	Oshita (2022)	^16^	55/71 [77%]		16/71 [23%]		ns	–	–	–	–
	Takeuchi (2022)	^71^	88/101 [87%]		13/101 [13%]		13/101 [13%]	–	–	–	–
	Kobayashi (2022)	^72^	ns		*ns*		16/127 [13%]	–	–	–	–
	Hirai (2022)	^73^	40/52 [77%] ^‡^		12/52 [23%] ^‡^		3/52 [6%]	–	–	–	–
	Banno (2022)	^74^	47/60 [78%]		13/60 [22%]		8/60 [13%]	–	–	–	–
	Matsuyama (2023)	^75^	ns		*ns*		18/109 [17%]	Placebo	*ns*	*ns*	17/70 [24%]
	Banno (2023)	^76^	51/67 [76%] ^‡^		16/67 [24%] ^‡^		8/67 [12%]	–	–	–	–
	Kagami (2023)	^77^	121/157 [77%] ^‡^		36/157 [23%] ^‡^		29/157 [18%]	–	–	–	–
	Ohtonari (2023)	^78^	34/41 [83%]		7/41 [17%]		ns	–	–	–	–
	Kobayashi (2023)	^79^	ns		ns		ns	–	–	–	–
**Condoliase (total)**			**722/919 [78%]**		**198/920 [22%]**		**135/1001 [13.4%]**	**–**	**–**	**–**	**–**
Collagenase	Sussman (1981)	^48^	25/29 [86%]		4/29 [14%]		4/29 [14%]	–	–	–	–
	Bromley (1982)	^80^	70/82 [85%]		12/82 [15%]		10/82 [12%]	–	–	–	–
	Bromley (1983)	^49^	40/46 [87%]		6/46 [13%]		6/46 [13%]	–	–	–	–
	Brown (1985)	^42^	34/54 [63%]		20/54 [37%]		13/54 [24%]	–	–	–	–
	Brown (1989) *	^50^	10/15 [67%]		5/15 [33%]		6/15 [40%]	Surgery ^‹^	18/19 [95%]	1/19 [5%]	0/19 [0%]
	Zhang (2015)	^81^	208/236 [88%]		28/236 [12%]		ns	–	–	–	–
	Wang (2021)	^17^	110/126 [87%] ^†^		16/126 [13%] ^†^		9/126 [7%]	–	–	–	–
	Wittenberg (2001) *	^32^	26/50 [52%]		24/50 [48%]		14/50 [28%]	–	–	–	–
**Collagenase (total)**			**523/638 [82%]**		**115/638 [18%]**		**62/402 [15.4%]**	**–**	**–**	**–**	**–**
**Chemonucleolysis (total)**			**6030/7588 [79%]**		**1559/7589 [21%]**		**743/6009 [12.4%]**	**–**	**–**	**–**	**–**
							**Surgery (total)**		**303/383 [79%]**	**80/383 [21%]**	**31/402 [7.7%]**
							**Placebo (total)**		**135/269 [50%]**	**134/269 [50%]**	**61/269 [22.7%]**
							**Combined (total)**		**438/652 [67%]**	**214/652 [33%]**	**92/671 [13.7%]**

Cohort summaries (cumulative totals) are in bold.

*Study involving a group of collagenase and a group of chymopapain-treated patients , #self-declared improvement, †as defined by Modified MacNab criteria, ‡Based on > 50% pain score (leg or back) improvement compared to baseline, ˆBased on > 20 mm VAS change, ‹Surgery-cohort applied in both collagenase and chymopapain from Brown et al. (1989).

Abbreviations: (L)—Low dose, (M)—Medium dose, (H)—High dose, ns—not specified.

### Risk of proceeding to surgery

From 46 articles including 6009 chemonucleolysis-treated LDH patients, 743 (12.4%) were reported to proceed to surgical alternatives after a variable period of time due to unsolved or recurring symptoms (Table 3, supplementary item 7). Rates were 11.9% and 13.4% for chymopapain and condoliase respectively, and slightly higher at 15.4% for collagenase-treated cohorts. In 6 studies^42,44,50,53,62,64^ comparing chemonucleolysis vs. surgical decompression, the pooled analysis of patients requiring further surgery due to failure of the index procedure did not show any significant difference (OR: 0.87, 95% CI: 0.42–7.41, *p* = 0.44; Fig. 3). Similar results were found following meta-analysis of the 6 studies^25,27,28,34,35,75^ comparing chemonucleolysis vs. placebo procedures (OR: 0.71, 95% CI: 0.35–1.44, *p* = 0.34; Fig. 3).

**Table 3:** Tabular overview of reported adverse events and the rate of patients proceeding to surgery.

	Article	Refs		Patient with SAE (n/N [%])	Infectious SAE (n/N [%])	Allergy prevention	Allergic reaction (n/N [%])	Anaphylactic shock (n/N [%])		Control number of SAE (n/N [%])
Chymopapain	Smith (1967)	^52^		3/75 [4%]	0/150 [0%]	ns	ns	1/75 [1%]		–
	Nordby (1972)	^53^		0/100 [0%]	1/45 [2%]	ns	ns	ns		3/91 [3%]
	Schwetschenau (1976)	^35^		0/31 [0%]	0/16 [0%]	H1 blocker, corticosteroid, antimuscarinic	0/31 [0%]	0/31 [0%]		0/35 [0%]
	Maroon (1976)	^36^		4/48 [8%]	2/51 [4%]	Corticosteroids	2/48 [4%]	0/48 [0%]		–
	Ravichandran (1980)	^37^		0/26 [0%]	ns	ns	2/26 [8%]	0/26 [0%]		–
	Javid (1983)	^25^		0/55 [0%]	0/60 [0%]	ns	0/55 [0%]	0/55 [0%]		1/53 [2%]
	Hall (1983)	^54^		15/4282 [0%]	1/30 [3%]	ns	ns	15/4282 [0%]		–
	Ejeskar (1983)	^26^		ns	ns	ns	0/15 [0%]	0/15 [0%]		ns
	Parkinson (1983)	^55^		0/33 [0%]	0/106 [0%]	None	0/33 [0%]	0/33 [0%]		–
	Sutton (1985a)	^56^		3/33 [9%]	ns	H1/H2 blockers	2/33 [6%]	1/33 [3%]		–
	Sutton (1985b)	^57^		17/189 [9%]	0/58 [0%]	ns	3/189 [2%]	1/189 [1%]		–
	Dabezies (1985)	^58^		ns	0/60 [0%]	ns	ns	ns		–
	Jabaay (1985)	^38^		ns	ns	ns	ns	ns		–
	McDermott (1985)	^39^		73/1498 [5%]	1/733 [0%]	H1/H2 blockers	55/1498 [4%]	14/1498 [1%]		–
	Javid (1985)	^59^		ns	0/42 [0%]	ns	ns	ns		–
	Lorenz (1985)	^60^		3/55 [5%]	1/84 [1%]	ns	1/55 [2%]	1/55 [2%]		–
	Nordby (1986)	^82^		3/748 [0%]	ns	ns	ns	2/748 [0%]		–
	Maciunas (1986)	^103^		0/268 [0%]	0/50 [0%]	ns	0/268 [0%]	0/268 [0%]		–
	Hill (1986)	^61^		2/335 [1%]	ns	H1/H2 blockers	ns	1/335 [0%]		–
	Dabezies (1987)	^27^		1/78 [1%]	0/48 [0%]	ns	2/78 [3%]	1/78 [1%]		0/81 [0%]
	Shields (1987)	^41^		1/150 [1%]	0/150 [0%]	H1/H2 blockers, corticosteroids (partly)	5/150 [3%]	0/150 [0%]		–
	Zeiger (1987)	^62^		1/45 [2%]	1/45 [2%]	ns	0/45 [0%]	0/45 [0%]		1/81 [1%]
	Hofstra (1989)	^63^		0/16 [0%]	0/16 [0%]	Corticosteroid	1/16 [6%]	0/16 [0%]		–
	Alexander (1989)	^64^		2/51 [4%]	2/51 [4%]	H1/H2 blockers	0/51 [0%]	0/51 [0%]		5/49 [10%]
	Brown (1989) *	^50^		ns	ns	ns	ns	ns		ns
	Boccanera (1990)	^43^		15/60 [25%]	0/60 [0%]	H1/H2 blockers	1/60 [2%]	0/60 [0%]		–
	Gogan (1991)	^28^		2/30 [7%]	1/30 [3%]	ns	0/30 [0%]	0/30 [0%]		6/30 [20%]
	LeBlanc (1991)	^65^		1/50 [2%]	ns	H1/H2 blockers	1/50 [2%]	0/50 [0%]		–
	Javid (1992)	^44^		0/106 [0%]	0/106 [0%]	H1 blocker	1/106 [1%]	0/106 [0%]		0/72 [0%]
	Kato (1992)	^45^		ns	ns	ns	ns	ns		–
	Benoist (1993) (L)	^31^		1/58 [2%]	0/58 [0%]	H1 blocker, antimuscarinic	2/58 [3%]	1/58 [2%]		–
	Benoist (1993) (M)			0/60 [0%]	0/60 [0%]	H1 blocker, antimuscarinic	1/60 [2%]	0/60 [0%]		–
	Kato (1993)	^66^		ns	ns	ns	ns	ns		–
	Leonardi (1993)	^84^		1/733 [0%]	1/733 [0%]	H1/H2 blockers, complement blockers	6/733 [1%]	0/733 [0%]		–
	Benoist (1993	^46^		0/42 [0%]	0/42 [0%]	ns	0/42 [0%]	0/42 [0%]		–
	Louwaege (1996	^83^		1/84 [1%]	1/84 [1%]	H1/H2 blockers , corticosteroids	0/84 [0%]	0/84 [0%]		–
	Leivseth (1999	^67^		ns	ns	ns	ns	ns		–
	Wittenberg (2001 *	^32^		1/50 [2%]	0/50 [0%]	H1/H2 blockers, corticosteroids	6/50 [12%]	0/50 [0%]		–
	Wardlaw (2013	^29^		ns	ns	ns	ns	ns		ns
	Wardlaw (2013	^30^		1/48 [2%]	0/48 [0%]	ns	ns	0/48 [0%]		1/52 [2%]
**Chymopapain (total)**				**151/9437 [1.6%]**	**9/4508 [0.2%]**	**–**	**91/3864 [2.4%]**	**38/9352 [0.4%]**		**–**
Condoliase	Matsuyama (2018) (L)		^33^	0/49 [0%]	0/49 [0%]	ns	2/49 [4%]	0/49 [0%]		2/47 [4%]
	(M)			0/49 [0%]	0/49 [0%]	ns	1/49 [2%]	0/49 [0%]		–
	(H)			0/49 [0%]	0/49 [0%]	ns	1/49 [2%]	0/49 [0%]		–
	Chiba (2018)		^34^	4/82 [5%]	0/82 [0%]	ns	4/82 [5%]	0/82 [0%]		6/81 [7%]
	Ishibashi (2020)		^68^	ns	ns	ns	ns	ns		–
	Nakajima (2020)		^69^	0/42 [0%]	0/42 [0%]	ns	ns	0/42 [0%]		–
	Okada (2020)		^15^	0/82 [0%]	0/82 [0%]	ns	3/82 [4%]	0/82 [0%]		–
	Inoue (2021)		^47^	2/84 [2%]	0/84 [0%]	ns	3/84 [4%]	0/84 [0%]		–
	Banno (2021)		^51^	0/47 [0%]	0/47 [0%]	ns	1/47 [2%]	0/47 [0%]		–
	Oshita (2022)		^16^	ns	ns	ns	ns	ns		–
	Takeuchi (2022)		^71^	0/101 [0%]	0/101 [0%]	ns	5/101 [5%]	0/101 [0%]		–
	Kobayashi (2022)		^72^	0/127 [0%]	0/127 [0%]	ns	3/127 [2%]	0/127 [0%]		–
	Hirai (2022)		^73^	0/52 [0%]	0/52 [0%]	ns	1/52 [2%]	0/52 [0%]		–
	Banno (2022)		^74^	0/60 [0%]	0/60 [0%]	ns	4/60 [7%]	0/60 [0%]		–
	Matsuyama (2023)		^75^	ns	ns	ns	ns	ns		ns
	Banno (2023)		^76^	0/67 [0%]	0/67 [0%]	ns	3/57 [5%]	0/67 [0%]		–
	Kagami (2023)		^77^	ns	ns	None	11/157 [7%]	0/157 [0%]		–
	Ohtonari (2023)		^78^	ns	ns	ns	ns	ns		–
	Kobayashi (2023)		^79^	0/26 [0%]	0/26 [0%]	ns	0/26 [0%]	0/26 [0%]		–
**Condoliase (total)**				**6/917 [0.7%]**	**0/917 [0.0%]**	**–**	**42/1022 [4.1%]**	**0/1074 [0.0%]**		**–**
C**ollagenase**	Sussman (1981)		^48^	0/29 [0%]	0/29 [0%]	ns	0/29 [0%]	0/29 [0%]		–
	Bromley (1982)		^80^	0/82 [0%]	0/82 [0%]	ns	ns	0/82 [0%]		–
	Bromley (1983)		^49^	0/52 [0%]	ns	ns	0/52 [0%]	0/52 [0%]		–
	Brown (1985)		^42^	1/54 [2%]	1/54 [2%]	H1 blocker, corticosteroid	ns	0/54 [0%]		–
	Brown (1989) *		^50^	ns	ns	ns	ns	ns		ns
	Zhang (2015)		^81^	0/236 [0%]	0/236 [0%]	ns	ns	0/236 [0%]		–
	Wang (2021)		^17^	1/126 [1%]	0/126 [0%]	ns	0/126 [0%]	0/126 [0%]		–
	Wittenberg (2001) *		^32^	0/50 [0%]	0/50 [0%]	H1/H2 blockers, corticosteroids	0/50 [0%]	0/50 [0%]		–
**Collagenase (total)**				**2/629 [0.3%]**	**1/577 [0.2%]**	**–**	**0/257 [0.0%]**	**0/629 [0.0%]**		**–**
**Chemonucleolysis (total)**				**159/10,983 [1.4%]**	**10/6002 [0.2%]**	**–**	**133/5143 [2.6%]**	**38/11055[0.3%]**		**–**
							**Surgery (total)**		**16/426 [3.8%]**	
							**Placebo/Sham (total)**		**9/246 [3.7%]**	
							**Control (total)**		**25/672 [3.7%]**	

Values are given as the number of events (n) within the patient population (N) and the corresponding percentage (%). Cohort summaries (cumulative totals) are in bold.

*Study involving a group of collagenase and a group of chymopapain injections.

Abbreviations: ns—not specified, SAE—Severe adverse events.

### Occurrence of SAEs

The rate of SAE was reported in 48 studies and showed 159/10,983 (1.4%) treated patients with one or more SAEs (Table 3, supplemental item 8). Notably, rates were slightly higher in chymopapain cohorts (1.6%) than in condoliase- (0.7%) and collagenase- (0.3%) treated cohorts. Five included studies^29,44,53,62,64^ examined the rate of SAEs in patients treated with chemonucleolysis vs. surgical decompression and 6 studies^25,27,28,34,35,75^ compared chemonucleolysis with placebo injections. In both cases, chemonucleolysis was not associated with a significantly higher rate of SAEs compared to both surgery (OR: 0.44, 95% CI: 0.15–1.34, *p* = 0.15; supplemental item 9) and placebo treatments (OR: 0.49, 95% CI: 0.21–1.15, *p* = 0.10; supplemental item 10).

SAEs were predominantly categorized as infectious, anaphylactic, or involving a severe worsening of LBP or disc features. Notably, infectious SAEs were almost exclusively observed in chymopapain-treated cohorts, accounting for 0.2% of cases, while only a single infection was reported in collagenase-treated patients (Table 3). Similarly, the occurrence of anaphylactic shock was solely documented in chymopapain-treated cohorts, affecting 38/9352 treated patients (0.4%; Table 3, supplemental item 11).

Interestingly, the prevalence of allergic reactions varied among different chemonucleolytic agents. Condoliase-treated cohorts exhibited a slightly higher incidence of allergic reactions, such as rashes, affecting 4.1% of patients, in contrast to 2.4% in chymopapain-treated cohorts and 0.0% in collagenase-treated cohorts (Table 3, supplementary item 11). Notably, it is important to highlight the absence of detailed specifications regarding preventative allergy suppressant medication in the condoliase group, while most studies in the chymopapain cohorts clearly implemented such preventative measures.

## Discussion

Chemonucleolysis has a historical context rooted in the pursuit of alternatives to conventional surgical interventions for LDH, with chymopapain being an early but subsequently discontinued option due to safety concerns^11^. Our findings shed light on the evolving landscape of chemonucleolysis, encompassing newer agents like condoliase and re-explored interest in collagenase-based therapies. Here our meta-analysis showed that chemonucleolysis significantly outperforms placebo in terms of treatment success, demonstrating its efficacy in alleviating LDH-associated radiculopathy and disability. The comparable rates of treatment success between chemonucleolysis and surgical intervention suggests that chemonucleolysis may be an effective standalone intervention that can be considered as an alternative to surgical interventions.

Concerns about SAEs (particularly anaphylactic shock) associated with chemonucleolysis were addressed in our analysis. While chymopapain exhibited a higher rate of severe anaphylactic reactions and infectious SAEs, condoliase and collagenase treatments demonstrated safer profiles, with no reported anaphylactic shocks and lower overall SAE rates. No prophylactic allergy treatments are provided with condoliase administration in Japan and none of the included studies provided detailed specifications regarding allergy suppressant medication. The potential benefit of such regimen is a possible avenue of research to potentially further enhance its safety profile. Condoliase, with its substrate-specificity for chondroitin sulfate and hyaluronic acid, presents a more targeted enzymatic alternative, potentially minimizing off-target effects observed with earlier chemonucleolytic agents. The exceptional specificity of condoliase for chondroitin sulfate, renders it an exceptionally selective agent within the intervertebral disc space^13,14^. This specificity stands in contrast to chymopapain or collagenase, as illustrated in Fig. 1C, emphasizing the expected superior safety profile associated with condoliase. Furthermore, condoliase may have additional implications, as it may simultaneously aid in nerve and spinal cord repair. Chondroitin sulfate is produced in response to damage in nerve tissue and hinders nerve growth and axon proliferation. Notably, chondroitinase ABC, a product of condoliase, has demonstrated efficacy in promoting functional recovery following spinal cord injury^86^. This dual capability of selectively targeting LDH-related pathology while potentially supporting neural repair underlines its potential therapeutic scope.

Despite promising observations, it is imperative to acknowledge the limitations of the current body of evidence, warranting a call for further comprehensive studies. The controlled studies included in this review, while providing valuable insights, exhibit certain drawbacks. The majority, especially those pertaining to chymopapain, are dated, with a considerable proportion conducted before the 2000s. Furthermore, a prevalent retrospective nature, relatively small cohort sizes, and high-risk of bias scores contribute to the overall limitations. Additionally, the heterogeneity in outcome measures and patient indications across studies, particularly in comparisons involving various chemonucleolytic agents, further underscores the need for caution in drawing definitive conclusions. To advance our understanding of the potential of chemonucleolytic enzymes, future research endeavors should prioritize the design and implementation of higher-quality trials. Large randomized controlled trials, characterized by rigorous methodologies and standardized reporting, hold the potential to offer more robust and generalizable insights into the efficacy and safety of these enzymatic chemonucleolytic interventions for LDH.

Moreover, further optimizations of chemonucleolysis techniques and study designs are warranted. For example, optimal patient selection criteria, considering factors like age, Pfirrmann grade^22^, and type of LDH have been suggested to enhance treatment outcomes^13,15,16,69^. Other aspects such as the type of contrast agent used, have also been shown to potentially influence enzymatic activity^87^. In addition, there is very little literature reporting long-term imaging evaluations of discs treated with chemonucleolysis (Supplemental item 4), and long-term clinical outcomes and more objective assessments remain largely undetermined^88^. Therefore, future work may consider revising the enzymatic destruction of herniated disc tissue through supplementation with regenerative treatments^16,89,90^ e.g., co-injection of cells^91,92^, biomaterials^93,94^, extracellular vesicles^95,96^, or platelet-rich plasma (PRP)^97^ to support the restoration of the disc over time, particularly regarding younger patients^16,79^. For example, previous animal studies have shown the capacity of PRP products to reverse condoliase-mediated disc deterioration^98^. Here, longer follow-ups are critical to fully grasp the impact of the enzymatic digestion of disc tissue on long-term spinal health^16^. Alternative chemonucleolysis methods, not involving enzymatic digestion, such as ethanol^99^ or ozone^100^ are also being explored and future reviews should seek to compare efficacy and safety outcomes of these approaches to enzymatic chemonucleolysis-treated LDH.

## Conclusion

Our comprehensive analysis underscores the evolving landscape of chemonucleolysis as a viable non-surgical intervention for LDH. With newer agents like condoliase exhibiting enhanced safety profiles and promising efficacy, the reevaluation of chemonucleolytic enzymes offers a valuable therapeutic avenue. Further research, particularly large randomized controlled trials, is imperative to solidify the evidence base and refine the clinical application of these enzymatic interventions, fostering their broader adoption in LDH management. We advocate for wider adoption and thorough evaluation of these techniques in clinical settings, particularly in Europe and the USA, to validate and refine their potential and determine the place of chemonucleolysis in the clinical toolbox to mend LDH.

### Supplementary Information

Supplementary Information.

## Data Availability

All data produced as part of this review are available in the main text or as a supplementary file. Additional requests can be made to the corresponding author upon reasonable request.
