# Supplementary material for: Enzymatic chemonucleolysis for lumbar disc herniation—an assessment of historical and contemporary efficacy and safety: a systematic review and meta-analysis
Source: Sci Rep. 2024 Jun 4;14:12846. doi: 10.1038/s41598-024-62792-8 (PMC11150519; doi:10.1038/s41598-024-62792-8)

**SUPPLEMENTAL ITEMS**

# Enzymatic Chemonucleolysis for Lumbar Disc Herniation – Assessment of historical and contemporary efficacy and safety: a Systematic Review and Meta-Analysis

Jordy Schol, Luca Ambrosio, Shota Tamagawa, et al.

**Supplemental item 1.** Overview of applied search syntax.

***PubMed***

("Chondroitin"[All Fields] OR "Chondroitin-sulfate"[All Fields] OR "Chondroitinase ABC"[All Fields] OR "Chondroitin sulfate"[All Fields] OR "Chondroitin sulphate"[All Fields] OR "Condoliase"[All Fields] OR "chymopapain"[All Fields] OR "chemonucleolysis"[All Fields] OR "Lyase"[All Fields] OR "endolyase"[All Fields] OR "collagenase"[All Fields] OR "nucleolysis"[All Fields] OR "Chondroitin ABC Lyase"[Mesh] OR "Chondroitinases and Chondroitin Lyases"[Mesh] OR "Chondroitin Lyases"[Mesh] OR "Intervertebral Disc Chemolysis"[Mesh]) AND ("lumbar" AND "herniation"[All Fields] OR "hernia"[All Fields] OR "spine"[All Fields] OR "spinal"[All Fields] OR "Intervertebral Disc Displacement"[Mesh] OR "Intervertebral Disc"[Mesh] OR "Sciatica"[Mesh]) AND ("human"[All Fields] OR "clinical"[All Fields] OR "clinic"[All Fields] OR "trial"[All Fields])

***Scopus***

( ALL ( "Chondroitin" ) OR ALL ( "Chondroitin-sulfate" ) OR ALL ( "Chondroitinase ABC" ) OR ALL ( "Chondroitin sulfate" ) OR ALL ( "Chondroitin sulphate" ) OR ALL ( "Condoliase" ) OR ALL ( "chymopapain" ) OR ALL ( "chemonucleolysis" ) OR ALL ( "Lyase" ) OR ALL ( "endolyase" ) OR ALL ( "collagenase" ) OR ALL ( "nucleolysis" ) OR ALL ( "Chondroitin ABC Lyase" ) OR ALL ( "Chondroitin Lyases" ) OR ALL ( "Intervertebral Disc Chemolysis" ) ) AND "lumbar" AND ( ALL ( "herniation" ) OR ALL ( "hernia" ) OR ALL ( "spine" ) OR ALL ( "spinal" ) OR ALL ( "Intervertebral Disc Displacement" ) OR ALL ( "Intervertebral Disc" ) OR ALL ( "Sciatica" ) ) AND ( ALL ( "human" ) OR ALL ( "clinical" ) OR ALL ( "clinic" ) OR ALL ( "trial" ) ) AND NOT ( "in vitro" ) AND NOT ( "preclinical" ) AND NOT ( "animal" ) AND NOT ( "case report" ) AND NOT ( "basic research" ) AND ( LIMIT-TO ( SRCTYPE , "j" ) ) AND ( LIMIT-TO ( DOCTYPE , "ar" ) ) AND ( LIMIT-TO ( SUBJAREA , "MEDI" ) ) AND ( LIMIT-TO ( LANGUAGE , "English" ) ) AND ( LIMIT-TO ( EXACTKEYWORD , "Human" ) )

**Supplemental item 2.** Tabular overview of patient cohorts and specified indications; sorted on the enzyme type being examined. * Study involving a group of collagenase and a group of chymopapain injections. 0 (red) stands for “no” or specified as exclusion, - stands for “unspecified” or “unclear”, 1 (green) stands for “yes” or specified as inclusion criteria. Abbreviation: Avg. – Average, CT – Computed tomography, FUMAX – Maximum follow-up, LDH – Lumbar disc herniation, MRI – Magnetic resonance imaging, ns – Not specified

| | | Trial design | | | | | | | | Patient indication | | | | | | | | | | | | | |
|---|---|---|---|---|---|---|---|---|---|---|---|---|---|---|---|---|---|---|---|---|---|---|---|
| | Article | Prospective | Blind: Patient | Blind: Assessor | Blind: Practitioner | Randomized | Controlled | Placebo | Crossover | Sciatica | Radicular /Leg pain | Discography | Myelography | CT confirmed | MRI confirmed | High-intensity zones | Protrusion | Extrusion | Subligamentous | Transligamentous | Sequestered | Repeated injection? | Multiple levels |
| Chymopapain | Smith, 1967 | 0 | 0 | 0 | 0 | 0 | 0 | 0 | 0 | 1 | 1 | 1 | 1 | 0 | 0 | - | - | - | - | - | - | 1 | 1 |
| | Nordby, 1972 | 0 | 0 | 0 | 0 | 0 | 0 | 0 | 0 | 1 | 1 | 1 | 1 | 0 | 0 | - | - | - | - | - | - | 0 | 0 |
| | Schwetschenau, 1976 | 1 | 1 | 1 | 1 | 1 | 1 | 1 | 0 | 1 | 1 | 0 | 1 | 0 | 0 | 0 | 1 | 1 | - | - | 1 | 0 | 1 |
| | Maroon, 1976 | 1 | 0 | 0 | 0 | 0 | 0 | 0 | 0 | 1 | 1 | 1 | 1 | - | - | - | - | - | - | - | - | - | 0 |
| | Ravichandran, 1980 | 1 | 0 | 0 | 0 | 0 | 0 | 0 | 0 | 1 | 1 | - | 1 | 0 | 0 | 0 | 1 | - | - | - | - | 0 | 1 |
| | Javid, 1983 | 1 | 1 | 1 | 1 | 1 | 1 | 1 | 1 | 1 | 1 | 1 | 1 | 0 | 0 | 0 | - | - | - | - | - | 0 | 0 |
| | Hall, 1983 | 0 | 0 | 0 | 0 | 0 | 0 | 0 | 0 | 1 | 1 | - | - | - | - | - | - | - | - | - | - | 0 | 1 |
| | Ejeskar, 1983 | 1 | 0 | 1 | 0 | 1 | 1 | 0 | 1 | 1 | 1 | 0 | 1 | 0 | 0 | - | - | - | - | - | - | 0 | 0 |
| | Parkinson, 1983 | 0 | 0 | 0 | 0 | 0 | 0 | 0 | 0 | - | 1 | - | 1 | - | - | - | 1 | - | - | - | - | - | - |
| | Sutton, 1985a | 0 | 0 | 0 | 0 | 0 | 0 | 0 | 0 | 1 | 1 | 1 | 1 | 1 | 0 | - | - | - | - | - | - | 1 | 1 |
| | Sutton, 1985b | 0 | 0 | 0 | 0 | 0 | 0 | 0 | 0 | - | - | 1 | - | - | - | - | - | - | - | - | - | 0 | 1 |
| | Dabezies, 1985 | 0 | 0 | 0 | 0 | 0 | 0 | 0 | 0 | - | - | 1 | 1 | 1 | 0 | - | - | - | - | - | - | 0 | 1 |
| | Jabaay, 1985 | 1 | 0 | 0 | 0 | 0 | 0 | 0 | 0 | 1 | 1 | 0 | 1 | 0 | 0 | - | - | - | - | - | - | - | 0 |
| | McDermott, 1985 | 1 | 0 | 0 | 0 | 0 | 0 | 0 | 0 | 1 | 1 | 1 | 1 | 0 | 0 | - | - | - | - | - | - | 0 | 1 |
| | Javid, 1985 | 0 | 0 | 0 | 0 | 0 | 0 | 0 | 0 | 1 | 1 | - | 1 | 0 | 0 | - | - | - | - | - | - | 0 | 1 |
| | Lorenz, 1985 | 0 | 0 | 0 | 0 | 0 | 0 | 0 | 0 | 1 | 1 | 0 | 1 | 0 | 0 | - | - | - | - | - | 1 | 0 | 1 |
| | Nordby, 1986 | 0 | 0 | 0 | 0 | 0 | 0 | 0 | 0 | 1 | 1 | 1 | 1 | 0 | 0 | 0 | - | - | - | - | - | - | 1 |
| | Maciunas, 1986 | 1 | 0 | 0 | 0 | 0 | 0 | 0 | 0 | 1 | 1 | 1 | 1 | 0 | 0 | - | - | - | - | - | - | 1 | 1 |
| | Hill, 1986 | 0 | 0 | 0 | 0 | 0 | 0 | 0 | 0 | 1 | 1 | 1 | 1 | 1 | 0 | - | - | - | - | - | - | 0 | 1 |
| | Dabezies, 1987 | 1 | 1 | 1 | 1 | 1 | 1 | 1 | 0 | 1 | 1 | 1 | 1 | 1 | 0 | 0 | - | - | - | - | - | 0 | 1 |
| | Shields, 1987 | 1 | 0 | 0 | 0 | 0 | 0 | 0 | 0 | 1 | 1 | 0 | 1 | 1 | 0 | 0 | 1 | 1 | - | - | - | 0 | 0 |
| | Zeiger, 1987 | 0 | 0 | 1 | 0 | 0 | 1 | 0 | 0 | 1 | 1 | 0 | 1 | 1 | 0 | 0 | - | - | - | - | - | 0 | 0 |
| | Hofstra, 1989 | 0 | 0 | 0 | 0 | 0 | 0 | 0 | 0 | 1 | 1 | 1 | 1 | 1 | - | - | - | - | - | - | - | 1 | 1 |
| | Alexander, 1989 | 0 | 0 | 0 | 0 | 0 | 1 | 0 | 0 | 1 | 1 | 1 | 1 | 1 | 0 | 0 | - | - | - | - | 1 | 0 | 1 |
| | Brown, 1989 | 1 | 0 | 0 | 0 | 0 | 1 | 0 | 0 | 1 | 1 | - | 1 | 1 | - | - | - | - | - | - | - | 0 | |
| | Boccanera, 1990 | 1 | 0 | 0 | 0 | 0 | 0 | 0 | 0 | 1 | 1 | 0 | 1 | 1 | 0 | 0 | - | - | - | - | - | 0 | 0 |
| | Gogan, 1991 | 1 | 1 | 1 | 1 | 1 | 1 | 1 | 0 | 1 | 1 | 1 | 1 | 0 | 0 | - | - | - | - | - | - | 1 | - |
| | LeBlanc, 1991 | 0 | 0 | 0 | 0 | 0 | 0 | 0 | 0 | 1 | 1 | 1 | - | - | 1 | - | - | - | - | - | 0 | - | - |
| | Javid, 1992 | 1 | 0 | 0 | 0 | 0 | 1 | 0 | 0 | 1 | 1 | 1 | 1 | 1 | 0 | 0 | 1 | 1 | - | - | 0 | 0 | 0 |
| | Kato, 1992 | 1 | 0 | 0 | 0 | 0 | 0 | 0 | 0 | 1 | 1 | 1 | 1 | 0 | 1 | 0 | - | - | - | - | - | 0 | 0 |
| | Benoist, 1993 | 1 | 1 | 1 | 1 | 1 | 1 | 0 | 0 | 1 | 1 | 1 | 0 | 1 | 1 | - | - | - | - | - | 0 | 0 | 0 |
| | Kato, 1993 | 0 | 0 | 0 | 0 | 0 | 0 | 0 | 0 | 1 | 1 | 1 | 1 | - | 1 | - | - | - | - | - | - | 0 | - |
| | Leonardi, 1993 | 0 | 0 | 0 | 0 | 0 | 0 | 0 | 0 | - | 1 | 1 | - | 1 | 0 | 0 | - | - | - | - | - | 0 | 1 |
| | Benoist, 1993 | 1 | 0 | 0 | 0 | 0 | 0 | 0 | 0 | 1 | 1 | 1 | 0 | 1 | 0 | 0 | - | - | - | - | - | 0 | 0 |
| | Louwaege, 1996 | 0 | 0 | 0 | 0 | 0 | 0 | 0 | 0 | 1 | 1 | 1 | - | 1 | 0 | - | - | - | - | - | - | - | - |
| | Leivseth, 1999 | 0 | 0 | 0 | 0 | 0 | 0 | 0 | 0 | 1 | 1 | - | - | 1 | 0 | - | - | - | - | - | 0 | 0 | - |
| | Wittenberg, 2001 | 1 | 0 | 0 | 0 | 1 | 1 | 0 | 0 | 1 | 1 | 1 | - | 1 | 1 | - | - | - | - | - | 0 | 0 | 1 |
| | Wardlaw, 2013a | 1 | 0 | 0 | 0 | 1 | 1 | 0 | 0 | 1 | 1 | - | 1 | - | 0 | 0 | - | - | - | - | - | 0 | - |
| | Wardlaw, 2013b | 1 | 0 | 0 | 0 | 1 | 1 | 0 | 0 | 1 | 1 | - | 1 | - | 0 | 0 | - | - | - | - | - | 0 | 0 |
| Condoliase | Matsuyama, 2018 | 1 | 1 | 1 | 1 | 1 | 1 | 1 | 0 | 1 | 1 | - | - | - | 1 | - | 1 | 1 | 1 | 0 | 0 | 0 | 0 |
| | Chiba, 2018 | 1 | 1 | 1 | 0 | 1 | 1 | 1 | 0 | - | 1 | - | - | - | 1 | - | 1 | 1 | 1 | 0 | 0 | 0 | - |
| | Ishibashi, 2020 | 0 | 0 | 0 | 0 | 0 | 0 | 0 | 0 | - | 1 | - | - | - | - | - | 1 | 1 | 1 | 1 | 0 | 0 | 0 |
| | Nakajima, 2020 | 0 | 0 | 0 | 0 | 0 | 0 | 0 | 0 | - | 1 | - | - | - | 1 | 1 | - | 1 | 1 | 1 | - | 0 | |
| | Okada, 2020 | 0 | 0 | 0 | 0 | 0 | 0 | 0 | 0 | - | 1 | - | - | - | 1 | - | - | 1 | 1 | 0 | 0 | 0 | |
| | Inoue, 2021 | 1 | 0 | 0 | 0 | 0 | 0 | 0 | 0 | - | 1 | - | - | - | 1 | - | - | 1 | 1 | 0 | 0 | 0 | |
| | Banno, 2021 | 1 | 0 | 0 | 0 | 0 | 0 | 0 | 0 | - | 1 | - | - | - | 1 | 1 | 1 | 1 | 1 | 1 | - | 0 | |
| | Oshita, 2022 | 0 | 0 | 0 | 0 | 0 | 0 | 0 | 0 | - | 1 | - | - | - | - | 1 | 1 | 1 | 1 | 1 | 0 | 0 | 0 |
| | Takeuchi, 2022 | 0 | 0 | 0 | 0 | 0 | 0 | 0 | 0 | 1 | 1 | - | - | - | 1 | 1 | - | - | - | - | - | 0 | 0 |
| | Kobayashi, 2022 | 0 | 0 | 0 | 0 | 0 | 0 | 0 | 0 | - | 1 | - | - | - | 1 | 1 | - | 1 | 1 | 1 | - | 0 | - |
| | Hirai, 2022 | 0 | 0 | 0 | 0 | 0 | 0 | 0 | 0 | - | 1 | - | - | - | 1 | - | - | 1 | 1 | 1 | 0 | 0 | |
| | Banno, 2022 | 0 | 0 | 0 | 0 | 0 | 0 | 0 | 0 | - | 1 | - | - | - | 1 | - | 1 | 1 | 1 | | | 0 | |
| | Matsuyama, 2023 | 0 | 0 | 0 | 1 | 1 | 1 | 1 | 0 | 1 | 1 | - | - | - | 1 | - | 1 | 1 | 1 | 0 | 0 | 0 | 0 |
| | Banno, 2023 | 0 | 0 | 0 | 0 | 0 | 0 | 0 | 0 | - | 1 | - | - | - | 1 | - | - | - | - | 0 | - | - | 0 |

| | | | | | | | | | | | | | | | | | | | | | | | |
|---|---|---|---|---|---|---|---|---|---|---|---|---|---|---|---|---|---|---|---|---|---|---|---|
| | Kagami, 2023 | 0 | 0 | 0 | 0 | 0 | 0 | 0 | 0 | - | 1 | - | - | - | 1 | - | - | 1 | 1 | 1 | - | 0 | 0 |
| | Ohtonari, 2023 | 0 | 0 | 0 | 0 | 0 | 0 | 0 | 0 | - | 1 | - | - | - | 1 | - | 0 | 1 | 1 | 0 | 0 | 0 | |
| | Kobayashi, 2023 | 1 | 0 | 0 | 0 | 0 | 0 | 0 | 0 | - | 1 | - | - | - | 1 | 1 | - | 1 | 1 | 1 | - | 0 | 0 |
| **Collagenase** | Sussman, 1981 | 0 | 0 | 0 | 0 | 0 | 0 | 0 | 0 | 1 | 1 | - | 1 | 0 | 0 | - | - | - | - | - | - | 0 | 0 |
| | Bromley, 1982 | 1 | 0 | 0 | 0 | 0 | 0 | 0 | 0 | 1 | 1 | 0 | 1 | - | - | - | - | - | - | - | - | 0 | 0 |
| | Bromley, 1983 | 1 | 0 | 0 | 0 | 0 | 0 | 0 | 0 | 1 | - | 0 | 1 | 0 | 0 | - | - | - | - | - | - | 0 | 0 |
| | Brown, 1985 | 1 | 0 | 0 | 0 | 0 | 1 | 0 | 0 | 1 | 1 | 1 | 1 | 1 | 0 | 0 | - | - | - | - | - | 1 | 1 |
| | Brown, 1989 | 0 | 0 | 0 | 0 | 0 | 0 | 0 | 0 | 1 | 1 | - | 1 | 1 | - | - | - | - | - | - | - | 0 | |
| | Zhang, 2015 | 0 | 0 | 0 | 0 | 0 | 0 | 0 | 0 | 1 | 1 | - | - | 1 | 1 | - | 1 | 1 | - | - | - | 0 | 0 |
| | Wang, 2021 | 1 | 0 | 0 | 0 | 1 | 1 | 0 | 0 | - | 1 | 1 | - | 1 | 1 | - | 0 | 0 | 0 | 0 | 1 | - | 1 |
| | Wittenberg, 2001 | 0 | 0 | 0 | 0 | 0 | 0 | 0 | 0 | 1 | 1 | 1 | - | 1 | 1 | - | - | - | - | - | 0 | 0 | 1 |

**Supplemental item 3.** Tabular overview of applied enzymatic chemonucleolytic products sorted by the type of enzyme applied. * Study involving a group of collagenase- and a group of chymopapain injections. Abbreviation: Adv. – Advance, Corp. – Corporation, Lab. – Laboratory, ns – not specified.

| Article | Product | Brand name (Manufacturer) | Volume | Concentration | Injected product | Needle gauge | Injection site |
|---|---|---|---|---|---|---|---|
| Smith, 1967 | Chymopapain | *ns* | *ns* | 4 mg/mL | *Variable* | *ns* | Intradiscal |
| Nordby, 1972 | Chymopapain | *ns* | 1.0-2.0 mL | 2000 U/mL | 2000-4000 U | *ns* | *ns* |
| Schwetschenau, 1976 | Chymopapain | Discase (Travenol Lab.) | 1.0 mL | 4 mg/mL | 4 mg | 22G | Intradiscal |
| Maroon, 1976 | Chymopapain | Discase (*ns*) | 1.0 mL | 2000 U/mL | 2000 U | 18G | Intradiscal |
| Ravichandran, 1980 | Chymopapain | Discase (*ns*) | *ns* | *ns* | *ns* | *ns* | *ns* |
| Javid, 1983 | Chymopapain | Chymodiactin | 1.5 mL | 2000 U/mL | 3000 U | 18G | Intradiscal |
| Hall, 1983 | Chymopapain | *ns* | *ns* | *ns* | *ns* | *ns* | Intradiscal |
| Ejeskar, 1983 | Chymopapain | Discase (Travenol Lab.) | 2.0 mL | 2000 U/mL | 4000 U | *ns* | Intradiscal |
| Parkinson, 1983 | Chymopapain | Discase (Travenol Lab.) | 1.0-2.0 mL | "1:2000" | *ns* | *ns* | Unspecified |
| Sutton, 1985a | Chymopapain | Discase (Travenol Lab.) & Chymodiactin (Flint Lab.) | *ns* | *ns* | *ns* | *ns* | Intradiscal |
| Sutton, 1985b | Chymopapain | Discase (Travenol Lab.) & Chymodiactin (Flint Lab.) | *ns* | *ns* | 2000-4000 U | 18G | Intradiscal |
| Dabezies, 1985 | Chymopapain | *ns* | *ns* | *ns* | *ns* | *ns* | *ns* |
| Jabaay, 1985 | Chymopapain | *ns* | *ns* | *ns* | *ns* | *ns* | Intradiscal |
| McDermott, 1985 | Chymopapain | Chymodiactin (Smith Lab.) | 1.5 mL | 2000 U/mL | 3000 U | 18G | Intradiscal |
| Javid, 1985 | Chymopapain | *ns* | *ns* | *ns* | 2000-4000 U | 18G | Intradiscal |
| Lorenz, 1985 | Chymopapain | *ns* | *ns* | *ns* | *ns* | *ns* | Intradiscal |
| Nordby, 1986 | Chymopapain | Discase | *ns* | *ns* | *ns* | *ns* | Intradiscal |
| Maciunas, 1986 | Chymopapain | *ns* | *ns* | *ns* | *ns* | *ns* | Intradiscal |
| Hill, 1986 | Chymopapain | Chymodiactin (Smith Lab.) | 1.7 mL | *ns* | *ns* | *ns* | Intradiscal |
| Dabezies, 1987 | Chymopapain | *ns* | 2.0 mL | 4 mg/mL | 8 mg | *ns* | Intradiscal |
| Shields, 1987 | Chymopapain | *ns* | 2.0 mL | *ns* | *ns* | *ns* | Intradiscal |
| Zeiger, 1987 | Chymopapain | Chymodiactin (Smith Lab.) | 2.5 mL | *ns* | *ns* | *ns* | Intradiscal |
| Hofstra, 1989 | Chymopapain | Discase (Travenol Lab.) & Chymodiactin (Flint Lab.) | *ns* | *ns* | 3000-4000 U | *ns* | Intradiscal |
| Alexander, 1989 | Chymopapain | *ns* | 1.4-1.8 mL | 2000 U/mL | 2800-3600 U | *ns* | Intradiscal |
| Brown, 1989 | Chymopapain * | *ns* | *ns* | *ns* | *ns* | *ns* | Intradiscal |
| Boccanera, 1990 | Chymopapain | Discase (Travenol Lab.) & Chymodiactin (Flint Lab.) | *ns* | *ns* | 0.5 nKat & 3000 U | *ns* | Intradiscal |
| Gogan, 1991 | Chymopapain | Discase (*ns)* | 2.0 mL | 4 mg/mL | 8 mg | *ns* | Intradiscal |
| LeBlanc, 1991 | Chymopapain | ns | *ns* | *ns* | *ns* | *ns* | *ns* |
| Javid, 1992 | Chymopapain | Chymodiactin (*ns*) | 1.5 mL | 2000 U/mL | 3000 U | *ns* | Intradiscal |
| Kato, 1992 | Chymopapain | Discase (Travenol Lab.) & Chymodiactin (Flint Lab.) | *ns* | *ns* | 5 nKat & 4 nKat | *ns* | Intradiscal |
| Benoist, 1993 | Chymopapain (Low dose) | Chymodiactin (Boots Pharma.) | 2.0 ml | 1000 U/mL | 2000 U | *ns* | Intradiscal |
| | Chymopapain (Medium dose) | Chymodiactin (Boots Pharma.) | 2.0 mL | 2000 U/mL | 4000 U | *ns* | Intradiscal |
| Kato, 1993 | Chymopapain | Discase (Travenol Lab.) & Chymodiactin (Flint Lab.) | *ns* | *ns* | 5 nKat & 4 nKat | *ns* | Intradiscal |
| Leonardi, 1993 | Chymopapain | *ns* | *ns* | *ns* | *ns* | *ns* | Intradiscal |
| Benoist, 1993 | Chymopapain | Chymodiactin (Smith Lab.) | *ns* | *ns* | 4000 U | *ns* | Intradiscal |
| Louwaege, 1996 | Chymopapain | Discase (Boots Pharma.) | 1.0-2.0 mL | *ns* | *ns* | 27G | Intradiscal |
| Leivseth, 1999 | Chymopapain | *ns* | 1.5-2.0 ml | 2000 pkat/mL | 3000-4000 pkat | *ns* | Intradiscal |
| Wittenberg, 2001 | Chymopapain * | *ns* | *ns* | *ns* | 4000 IU | *ns* | Intradiscal |
| Wardlaw, 2013a | Chymopapain | Chymodiactin (Smith Lab.) | *ns* | *ns* | *ns* | *ns* | Intradiscal |
| Wardlaw, 2013b | Chymopapain | Chymodiactin (Smith Lab.) | *ns* | *ns* | *ns* | *ns* | Intradiscal |
| Matsuyama, 2018 | Condoliase (Low dose) | Hernicore (Seikagaku Corp.) | 1.0 mL | 1.25 U/mL | 1.25 U | 21 - 23G | Intradiscal |
| | Condoliase (Medium dose) | | | 2.5 U/mL | 2.50 U | | |
| | Condoliase (High dose) | | | 5.0 U/mL | 5.00 U | | |
| Chiba, 2018 | Condoliase | *ns* | 1.0 mL | 1.25 U/mL | 1.25 U | 21 - 23G | Intradiscal |
| Ishibashi, 2020 | Condoliase | Hernicore (Seikagaku Corp.) | 1.0 mL | 1.25U / mL | 1.25 U | 22G | Intradiscal |
| Nakajima, 2020 | Condoliase | *ns* | 1.0 mL | 1.25 U/mL | 1.25 U | 21G | Intradiscal |
| Okada, 2020 | Condoliase | Hernicore (Seikagaku Corp.) | 1.0 mL | 1.25 U/mL | 1.25 U | 21G | Intradiscal |
| Inoue, 2021 | Condoliase | *ns* | *ns* | *ns* | *ns* | *ns* | Intradiscal |
| Banno, 2021 | Condoliase | *ns* | 1.0 mL | 1.25 U/mL | 1.25 U | 21G | Intradiscal |
| Oshita, 2022 | Condoliase | Hernicore (Seikagaku Corp.) | 1.0 mL | 1.25 U/mL | 1.25 U | *ns* | Intradiscal |
| Takeuchi, 2022 | Condoliase | Hernicore (Seikagaku Corp.) | 1.0 mL | 1.25 U/mL | 1.25 U | 22G | Intradiscal |
| Kobayashi, 2022 | Condoliase | *ns* | 1.0 mL | 1.25 U/mL | 1.25 U | *ns* | Intradiscal |
| Hirai, 2022 | Condoliase | Hernicore (Seikagaku Corp.) | 1.2 mL | 1.25 U/mL | 1.50 U | *ns* | Intradiscal |
| Banno, 2022 | Condoliase | *ns* | 1.0 mL | 1.25 U/mL | 1.25 U | *ns* | Intradiscal |
| Matsuyama, 2023 | Condoliase | Hernicore (Seikagaku Corp.) | 1.0 mL | 1.25 U/mL | 1.25 U | *ns* | Intradiscal |
| Banno, 2023 | Condoliase | *ns* | 1.0 mL | 1.0 U/mL | 1.00 U | 21G | Intradiscal |
| Kagami, 2023 | Condoliase | *ns* | 1.0 mL | 1.25 U/mL | 1.25 U | 21G | Intradiscal |
| Ohtonari, 2023 | Condoliase | *ns* | 1.2 mL | 1.04 U/mL | 1.25 U | 21G | Intradiscal |
| Kobayashi, 2023 | Condoliase | *ns* | 1.0 mL | 1.25 U/mL | 1.25 U | *ns* | Intradiscal |
| Sussman, 1981 | Collagenase | Nucleolysin (Adv. Biofactures Corp.) | *ns* | *ns* | 300 - 600 U | 22G | Intradiscal |
| Bromley, 1982 | Collagenase | *ns* | *ns* | *ns* | 300 - 600 U | 18G or 20G | Intradiscal |
| Bromley, 1983 | Collagenase | Nucleolysin (Adv. Biofactures Corp.) | 1.0 mL | 300 - 600 U/mL | 300 - 600 U | *ns* | *ns* |
| Brown, 1985 | Collagenase | Nucleolysin (Adv. Biofactures Corp.) | *ns* | *ns* | 600 U | *ns* | Intradiscal |
| Brown, 1989 | Collagenase * | *ns* | *ns* | *ns* | 600 U | *ns* | Intradiscal |
| Zhang, 2015 | Collagenase | *ns* | 0.3-0.5 mL | 75-200 U/0.3-0.5 ml | 75-200 U | *ns* | Intradiscal |
| Wang, 2021 | Collagenase | *ns* (Liaoning Wei Bang) | 0.1-0.4 mL | 125 - 2000 U /mL | 5 - 80 U | *ns* | Intradiscal |
| Wittenberg, 2001 | Collagenase * | *ns* | *ns* | *ns* | *ns* | *ns* | Intradiscal |

**Supplemental item 4.** Tabular overview of reported imaging outcomes. Values represent average values reported. Abbreviation: DH – Disc height, DHI – Disc height index, HV – Hernia volume.

| Article | Product | Type | Baseline (treatment) | 3M (treatment) | 12M (treatment) | Baseline (control) | 3M (control) | 12M (control) |
|---|---|---|---|---|---|---|---|---|
| Matsuyama, 2018 | Condoliase (L dose) | DH | 0.26 | 0.22 | - | 0.26 | 0.25 | - |
| | Condoliase (M dose) | DH | 0.26 | 0.21 | - | - | - | - |
| | Condoliase (H dose) | DH | 0.26 | 0.21 | - | - | - | - |
| Chiba, 2018 | Condoliase | DH | 0.26 | 0.22 | 0.21 | 0.27 | 0.26 | 0.21 |
| Banno, 2023 | Condoliase | DH | 8.2 | 6.7 | 7.4 | - | - | - |
| Hirai, 2022 | Condoliase | DH | 7.9 | 6.4 | - | - | - | - |
| Inoue, 2021 | Condoliase | %DHI | 100 | 86.8 | - | - | - | - |
| Kobayashi, 2023 | Condoliase | DHI | 27.4 | 16.8 | - | - | - | - |
| Chiba, 2018 | Condoliase | HV | 1.20 | 0.91 | 0.74 | 1.30 | 1.11 | 0.96 |
| Matsuyama, 2018 | Condoliase (L dose) | HV | 1.49 | 1.24 | - | 1.39 | 1.22 | - |
| | Condoliase (M dose) | HV | 1.22 | 0.98 | - | - | - | - |
| | Condoliase (H dose) | HV | 1.44 | 1.2 | - | - | - | - |
| Kagami, 2023 | Condoliase | Pfirrmann | 3.3 | 3.5 | - | - | - | - |
| Ohtonari, 2023 | Condoliase | Pfirrmann | 2.9 | 3.4 | - | - | - | - |

**Supplemental item 5.** Risk of bias assessment through the MINORS classification scheme and for randomized controlled trials also the Cochrane Source of bias tool.

| | MINORS | | | | | | | | | | | | | Cochrane Source of bias | | | | | | | | | | | | | |
|---|---|---|---|---|---|---|---|---|---|---|---|---|---|---|---|---|---|---|---|---|---|---|---|---|---|---|---|
| Article | A | B | C | D | E | F | G | H | I | J | K | L | Sum | A | B | C | D | E | F | G | H | I | J | K | L | M | Sum |
| Smith, 1967 | 1 | 2 | 2 | 1 | 0 | 1 | 2 | 0 | - | - | - | - | 9 | - | - | - | - | - | - | - | - | - | - | - | - | - | |
| Nordby, 1972 | 1 | 2 | 1 | 1 | 0 | 2 | 2 | 0 | - | - | - | - | 9 | - | - | - | - | - | - | - | - | - | - | - | - | - | |
| Schwetschenau, 1976 | 1 | 1 | 2 | 1 | 2 | 1 | 2 | 0 | 1 | 2 | 2 | 1 | 16 | 1 | 2 | 2 | 2 | 2 | 2 | 2 | 1 | 2 | 1 | 1 | 2 | 1 | 21 |
| Maroon, 1976 | 1 | 1 | 1 | 1 | 1 | 1 | 0 | 0 | - | - | - | - | 6 | - | - | - | - | - | - | - | - | - | - | - | - | - | |
| Ravichandran, 1980 | 0 | 1 | 1 | 0 | 0 | 2 | 0 | 0 | - | - | - | - | 4 | - | - | - | - | - | - | - | - | - | - | - | - | - | |
| Javid, 1983 | 2 | 2 | 2 | 2 | 2 | 1 | 2 | 2 | 1 | 2 | 2 | 2 | 22 | 2 | 2 | 2 | 2 | 2 | 2 | 2 | 2 | 2 | 2 | 1 | 2 | 1 | 24 |
| Hall, 1983 | 1 | 0 | 1 | 1 | 1 | 0 | 0 | 0 | - | - | - | - | 4 | - | - | - | - | - | - | - | - | - | - | - | - | - | |
| Ejeskar, 1983 | 1 | 2 | 2 | 1 | 2 | 2 | 2 | 0 | 1 | 2 | 1 | 0 | 16 | 1 | 1 | 1 | 0 | 2 | 2 | 0 | 1 | 1 | 1 | 0 | 0 | 1 | 11 |
| Parkinson, 1983 | 0 | 1 | 1 | 1 | 1 | 2 | 1 | 1 | - | - | - | - | 8 | - | - | - | - | - | - | - | - | - | - | - | - | - | |
| Sutton, 1985a | 2 | 2 | 0 | 2 | 0 | 2 | 2 | 0 | - | - | - | - | 10 | - | - | - | - | - | - | - | - | - | - | - | - | - | |
| Sutton, 1985b | 0 | 2 | 0 | 2 | 0 | 2 | 0 | 0 | - | - | - | - | 6 | - | - | - | - | - | - | - | - | - | - | - | - | - | |
| Dabezies, 1985 | 0 | 0 | 0 | 1 | 0 | 1 | 2 | 0 | - | - | - | - | 4 | - | - | - | - | - | - | - | - | - | - | - | - | - | |
| Jabaay, 1985 | 0 | 2 | 2 | 0 | 0 | 2 | 2 | 0 | - | - | - | - | 8 | - | - | - | - | - | - | - | - | - | - | - | - | - | |
| McDermott, 1985 | 1 | 2 | 2 | 2 | 0 | 2 | 0 | 0 | - | - | - | - | 9 | - | - | - | - | - | - | - | - | - | - | - | - | - | |
| Javid, 1985 | 0 | 1 | 0 | 2 | 0 | 2 | 0 | 0 | - | - | - | - | 5 | - | - | - | - | - | - | - | - | - | - | - | - | - | |
| Lorenz, 1985 | 1 | 2 | 0 | 1 | 0 | 1 | 2 | 0 | - | - | - | - | 7 | - | - | - | - | - | - | - | - | - | - | - | - | - | |
| Nordby, 1986 | 0 | 2 | 0 | 1 | 0 | 2 | 0 | 0 | - | - | - | - | 5 | - | - | - | - | - | - | - | - | - | - | - | - | - | |
| Maciunas, 1986 | 0 | 2 | 2 | 2 | 0 | 2 | 0 | 0 | - | - | - | - | 8 | - | - | - | - | - | - | - | - | - | - | - | - | - | |
| Hill, 1986 | 0 | 0 | 0 | 1 | 0 | 0 | 2 | 0 | - | - | - | - | 3 | - | - | - | - | - | - | - | - | - | - | - | - | - | |
| Dabezies, 1987 | 1 | 2 | 2 | 1 | 2 | 1 | 1 | 2 | 1 | 2 | 2 | 1 | 18 | 1 | 2 | 2 | 2 | 1 | 0 | 1 | 1 | 2 | 2 | 1 | 2 | 1 | 18 |
| Shields, 1987 | 1 | 2 | 2 | 2 | 0 | 2 | 0 | 0 | - | - | - | - | 9 | - | - | - | - | - | - | - | - | - | - | - | - | - | |
| Zeiger, 1987 | 1 | 2 | 2 | 2 | 1 | 2 | 2 | 0 | 1 | 1 | 0 | 0 | 14 | - | - | - | - | - | - | - | - | - | - | - | - | - | |
| Hofstra, 1989 | 1 | 1 | 1 | 1 | 0 | 2 | 1 | 0 | - | - | - | - | 7 | - | - | - | - | - | - | - | - | - | - | - | - | - | |
| Alexander, 1989 | 2 | 2 | 2 | 2 | 0 | 2 | 2 | 0 | 2 | 2 | 2 | 0 | 18 | - | - | - | - | - | - | - | - | - | - | - | - | - | |
| Brown, 1989 | 2 | 1 | 1 | 1 | 1 | 1 | 1 | 0 | 2 | 2 | 2 | 1 | 15 | - | - | - | - | - | - | - | - | - | - | - | - | - | |
| Boccanera, 1990 | 2 | 2 | 2 | 1 | 0 | 1 | 2 | 0 | - | - | - | - | 10 | - | - | - | - | - | - | - | - | - | - | - | - | - | |
| Gogan, 1991 | 2 | 2 | 1 | 2 | 2 | 2 | 2 | 0 | 1 | 2 | 1 | 0 | 17 | 1 | 2 | 2 | 2 | 2 | 2 | 2 | 2 | 1 | 1 | 2 | 2 | 1 | 22 |
| LeBlanc, 1991 | 0 | 0 | 1 | 1 | 1 | 1 | 1 | 0 | - | - | - | - | 5 | - | - | - | - | - | - | - | - | - | - | - | - | - | |
| Javid, 1992 | 1 | 2 | 2 | 1 | 1 | 2 | 1 | 0 | 2 | 2 | 1 | 1 | 16 | - | - | - | - | - | - | - | - | - | - | - | - | - | |
| Kato, 1992 | 1 | 1 | 2 | 1 | 0 | 2 | 2 | 0 | - | - | - | - | 9 | - | - | - | - | - | - | - | - | - | - | - | - | - | |
| Benoist, 1993 | 2 | 2 | 2 | 2 | 2 | 2 | 1 | 0 | 1 | 2 | 2 | 2 | 20 | 1 | 2 | 2 | 2 | 2 | 0 | 2 | 2 | 2 | 2 | 1 | 2 | 1 | 21 |
| Kato, 1993 | 2 | 1 | 1 | 1 | 0 | 2 | 0 | 0 | - | - | - | - | 7 | - | - | - | - | - | - | - | - | - | - | - | - | - | |
| Leonardi, 1993 | 0 | 2 | 1 | 1 | 0 | 0 | 2 | 0 | - | - | - | - | 6 | - | - | - | - | - | - | - | - | - | - | - | - | - | |
| Benoist, 1993 | 1 | 2 | 2 | 1 | 0 | 1 | 2 | 0 | - | - | - | - | 9 | - | - | - | - | - | - | - | - | - | - | - | - | - | |
| Louwaege, 1996 | 0 | 1 | 0 | 1 | 0 | 1 | 1 | 0 | - | - | - | - | 4 | - | - | - | - | - | - | - | - | - | - | - | - | - | |
| Leivseth, 1999 | 2 | 1 | 1 | 2 | 1 | 2 | 1 | 2 | - | - | - | - | 12 | - | - | - | - | - | - | - | - | - | - | - | - | - | |
| Wittenberg, 2001 | 1 | 0 | 2 | 1 | 1 | 2 | 0 | 0 | 1 | 2 | 1 | 1 | 12 | 1 | 1 | 1 | 1 | 1 | 1 | 1 | 1 | 1 | 1 | 1 | 1 | 1 | 13 |
| Wardlaw, 2013a | 2 | 2 | 1 | 2 | 1 | 2 | 1 | 0 | 2 | 2 | 0 | 1 | 16 | 1 | 1 | 1 | 1 | 1 | 1 | 2 | 1 | 1 | 2 | 1 | 2 | 1 | 16 |
| Wardlaw, 2013b | 1 | 2 | 1 | 2 | 1 | 2 | 1 | 0 | 2 | 2 | 0 | 1 | 15 | 1 | 1 | 1 | 1 | 1 | 1 | 2 | 1 | 1 | 2 | 1 | 2 | 1 | 16 |
| Matsuyama, 2018 | 2 | 1 | 2 | 2 | 2 | 2 | 1 | 2 | 1 | 2 | 1 | 2 | 20 | 2 | 2 | 2 | 2 | 2 | 0 | 2 | 2 | 2 | 2 | 1 | 2 | 1 | 22 |
| Chiba, 2018 | 1 | 2 | 2 | 2 | 2 | 2 | 2 | 2 | 1 | 2 | 2 | 2 | 22 | 1 | 2 | 2 | 1 | 1 | 2 | 0 | 2 | 2 | 2 | 2 | 2 | 1 | 20 |
| Ishibashi, 2020 | 1 | 1 | 1 | 1 | 1 | 1 | 1 | 0 | - | - | - | - | 7 | - | - | - | - | - | - | - | - | - | - | - | - | - | |
| Okada, 2020 | 1 | 1 | 1 | 2 | 1 | 2 | 1 | 0 | - | - | - | - | 9 | - | - | - | - | - | - | - | - | - | - | - | - | - | |
| Nakajima, 2020 | 1 | 2 | 1 | 1 | 1 | 1 | 2 | 0 | - | - | - | - | 9 | - | - | - | - | - | - | - | - | - | - | - | - | - | |
| Okada, 2020 | 1 | 2 | 1 | 1 | 1 | 2 | 2 | 0 | - | - | - | - | 10 | - | - | - | - | - | - | - | - | - | - | - | - | - | |
| Inoue, 2021 | 1 | 2 | 2 | 2 | 1 | 1 | 2 | 0 | - | - | - | - | 11 | - | - | - | - | - | - | - | - | - | - | - | - | - | |
| Banno, 2021 | 1 | 2 | 2 | 1 | 1 | 1 | 2 | 0 | - | - | - | - | 10 | - | - | - | - | - | - | - | - | - | - | - | - | - | |
| Oshita, 2022 | 2 | 0 | 1 | 2 | 1 | 1 | 1 | 0 | - | - | - | - | 8 | - | - | - | - | - | - | - | - | - | - | - | - | - | |
| Takeuchi, 2022 | 1 | 1 | 1 | 1 | 1 | 1 | 2 | 0 | - | - | - | - | 8 | - | - | - | - | - | - | - | - | - | - | - | - | - | |
| Kobayashi, 2022 | 2 | 1 | 1 | 1 | 2 | 1 | 0 | 0 | - | - | - | - | 8 | - | - | - | - | - | - | - | - | - | - | - | - | - | |
| Hirai, 2022 | 1 | 1 | 1 | 2 | 1 | 1 | 2 | 0 | - | - | - | - | 9 | - | - | - | - | - | - | - | - | - | - | - | - | - | |
| Banno, 2022 | 2 | 1 | 2 | 2 | 1 | 2 | 1 | 0 | - | - | - | - | 11 | - | - | - | - | - | - | - | - | - | - | - | - | - | |
| Matsuyama, 2023 | 2 | 1 | 2 | 2 | 1 | 2 | 1 | 0 | 1 | 1 | 2 | 2 | 17 | - | - | - | - | - | - | - | - | - | - | - | - | - | |
| Banno, 2023 | 1 | 1 | 1 | 2 | 1 | 2 | 1 | 0 | - | - | - | - | 9 | - | - | - | - | - | - | - | - | - | - | - | - | - | |
| Kagami, 2023 | 2 | 2 | 1 | 1 | 1 | 2 | 2 | 0 | - | - | - | - | 11 | - | - | - | - | - | - | - | - | - | - | - | - | - | |
| Ohtonari, 2023 | 1 | 2 | 1 | 2 | 1 | 1 | 2 | 0 | - | - | - | - | 10 | - | - | - | - | - | - | - | - | - | - | - | - | - | |
| Kobayashi, 2023 | 2 | 2 | 1 | 1 | 2 | 1 | 0 | 0 | - | - | - | - | 9 | - | - | - | - | - | - | - | - | - | - | - | - | - | |
| Sussman, 1981 | 0 | 1 | 2 | 1 | 1 | 1 | 2 | 0 | - | - | - | - | 8 | - | - | - | - | - | - | - | - | - | - | - | - | - | |
| Bromley, 1982 | 1 | 0 | 1 | 1 | 1 | 2 | 1 | 0 | - | - | - | - | 7 | - | - | - | - | - | - | - | - | - | - | - | - | - | |
| Bromley, 1983 | 0 | 1 | 2 | 1 | 0 | 1 | 0 | 0 | - | - | - | - | 5 | - | - | - | - | - | - | - | - | - | - | - | - | - | |
| Brown, 1985 | 1 | 0 | 2 | 1 | 1 | 2 | 2 | 0 | - | - | - | - | 9 | - | - | - | - | - | - | - | - | - | - | - | - | - | |
| Brown, 1989 | 2 | 1 | 2 | 1 | 1 | 1 | 1 | 0 | 2 | 2 | 2 | 1 | 16 | - | - | - | - | - | - | - | - | - | - | - | - | - | |
| Zhang, 2015 | 2 | 0 | 1 | 1 | 1 | 1 | 1 | 2 | - | - | - | - | 9 | - | - | - | - | - | - | - | - | - | - | - | - | - | |
| Wang, 2021 | 1 | 1 | 1 | 2 | 1 | 2 | 1 | 0 | - | - | - | - | 9 | - | - | - | - | - | - | - | - | - | - | - | - | - | |
| Wittenberg, 2001 | 1 | 0 | 2 | 1 | 1 | 2 | 0 | 0 | 1 | 2 | 1 | 1 | 12 | 1 | 1 | 1 | 1 | 1 | 1 | 1 | 1 | 1 | 1 | 1 | 1 | 1 | 13 |

**Supplemental item 6.** Single-arm meta-analysis of treatment success.

## Treatment success (Chemonucleolysis - Total)

| Study | Year | Events | Total | Weight | Proportion | 95% CI |
|---|---|---|---|---|---|---|
| Alexander | 1989 | 40 | 51 | 1.7% | 78.43 | [64.68; 88.71] |
| Banno | 2021 | 33 | 46 | 1.8% | 71.74 | [56.54; 84.01] |
| Banno | 2022 | 47 | 60 | 1.8% | 78.33 | [65.80; 87.93] |
| Banno | 2023 | 51 | 67 | 1.9% | 76.12 | [64.14; 85.69] |
| Benoist | 1993b | 33 | 42 | 1.6% | 78.57 | [63.19; 89.70] |
| Benoist (L) | 1993a | 36 | 41 | 1.4% | 87.80 | [73.80; 95.92] |
| Benoist (M) | 1993a | 38 | 43 | 1.4% | 88.37 | [74.92; 96.11] |
| Boccanera | 1990 | 54 | 60 | 1.5% | 90.00 | [79.49; 96.24] |
| Bromley | 1982 | 70 | 82 | 1.8% | 85.37 | [75.83; 92.20] |
| Bromley | 1983 | 40 | 46 | 1.5% | 86.96 | [73.74; 95.06] |
| Brown | 1989 | 35 | 51 | 1.8% | 68.63 | [54.11; 80.89] |
| Brown | 1985 | 34 | 54 | 1.9% | 62.96 | [48.74; 75.71] |
| Brown | 1989 | 10 | 15 | 1.2% | 66.67 | [38.38; 88.18] |
| Chiba | 2018 | 65 | 82 | 1.9% | 79.27 | [68.89; 87.43] |
| Dabezies | 1985 | 272 | 382 | 2.2% | 71.20 | [66.38; 75.70] |
| Dabezies | 1987 | 44 | 62 | 1.9% | 70.97 | [58.05; 81.80] |
| Ejeskar | 1983 | 13 | 15 | 0.9% | 86.67 | [59.54; 98.34] |
| Gogan | 1991 | 24 | 30 | 1.4% | 80.00 | [61.43; 92.29] |
| Hill | 1986 | 261 | 335 | 2.2% | 77.91 | [73.08; 82.24] |
| Hirai | 2022 | 40 | 52 | 1.8% | 76.92 | [63.16; 87.47] |
| Hofstra | 1989 | 15 | 15 | 0.3% | 100.00 | [78.20; 100.00] |
| Inoue | 2021 | 65 | 84 | 1.9% | 77.38 | [66.95; 85.80] |
| Ishibashi | 2020 | 21 | 34 | 1.7% | 61.76 | [43.56; 77.83] |
| Jaabay | 1985 | 221 | 263 | 2.1% | 84.03 | [79.03; 88.24] |
| Javid | 1983 | 40 | 55 | 1.8% | 72.73 | [59.04; 83.86] |
| Javid | 1985 | 85 | 105 | 2.0% | 80.95 | [72.13; 87.96] |
| Javid | 1992 | 90 | 104 | 1.9% | 86.54 | [78.45; 92.44] |
| Kagami | 2023 | 121 | 157 | 2.1% | 77.07 | [69.70; 83.39] |
| Kato | 1993 | 27 | 28 | 0.6% | 96.43 | [81.65; 99.91] |
| LeBlanc | 1991 | 37 | 50 | 1.8% | 74.00 | [59.66; 85.37] |
| Leonardi | 1993 | 602 | 733 | 2.3% | 82.13 | [79.16; 84.84] |
| Leviseth | 1999 | 24 | 50 | 1.9% | 48.00 | [33.66; 62.58] |
| Lorenz | 1985 | 44 | 55 | 1.7% | 80.00 | [67.03; 89.57] |
| Louwaege | 1996 | 61 | 84 | 2.0% | 72.62 | [61.80; 81.79] |
| Maciunas | 1986 | 230 | 268 | 2.1% | 85.82 | [81.06; 89.77] |
| Maroon | 1976 | 39 | 48 | 1.7% | 81.25 | [67.37; 91.05] |
| McDermott | 1985 | 1226 | 1402 | 2.3% | 87.45 | [85.60; 89.14] |
| Nakajima | 2020 | 32 | 42 | 1.7% | 76.19 | [60.55; 87.95] |
| Nordby | 1972 | 74 | 100 | 2.0% | 74.00 | [64.27; 82.26] |
| Nordby | 1986 | 493 | 739 | 2.3% | 66.71 | [63.18; 70.10] |
| Ohtonari | 2023 | 34 | 41 | 1.5% | 82.93 | [67.94; 92.85] |
| Okada | 2020 | 70 | 82 | 1.8% | 85.37 | [75.83; 92.20] |
| Oshita | 2022 | 55 | 71 | 1.9% | 77.46 | [66.00; 86.54] |
| Parkinson | 1983 | 198 | 200 | 0.9% | 99.00 | [96.43; 99.88] |
| Ravichandran | 1980 | 20 | 26 | 1.4% | 76.92 | [56.35; 91.03] |
| Schwetschenau | 1976 | 15 | 31 | 1.7% | 48.39 | [30.15; 66.94] |
| Shields | 1987 | 50 | 126 | 2.1% | 39.68 | [31.08; 48.78] |
| Smith | 1967 | 68 | 75 | 1.6% | 90.67 | [81.71; 96.16] |
| Sussman | 1981 | 25 | 29 | 1.2% | 86.21 | [68.34; 96.11] |
| Sutton | 1985a | 24 | 33 | 1.6% | 72.73 | [54.48; 86.70] |
| Sutton | 1985b | 147 | 189 | 2.1% | 77.78 | [71.17; 83.49] |
| Takeuchi | 2022 | 88 | 101 | 1.8% | 87.13 | [79.00; 92.96] |
| Wang | 2021 | 110 | 126 | 1.9% | 87.30 | [80.20; 92.56] |
| Wardlaw | 2013b | 42 | 45 | 1.1% | 93.33 | [81.73; 98.60] |
| Wittenberg | 2001a | 36 | 50 | 1.8% | 72.00 | [57.51; 83.77] |
| Wittenberg | 2001b | 26 | 50 | 1.9% | 52.00 | [37.42; 66.34] |
| Zeiger | 1987 | 27 | 45 | 1.8% | 60.00 | [44.33; 74.30] |
| Zhang | 2015 | 208 | 236 | 2.1% | 88.14 | [83.31; 91.97] |
| **Total (95% CI)** | | **6030** | **7588** | **100.0%** | **78.50** | **[75.42; 81.29]** |

Heterogeneity: $Tau^2 = 0.3387$; $Chi^2 = 415.50$, df = 57 ($P < 0.01$); $I^2 = 86\%$

Events per 100 observations, IV, Random, 95% CI — axis: 40 50 60 70 80 90 100; Treatment success rate (condoliase)

## Treatment success (Chymopapain)

| Study | Year | Events | Total | Weight | Proportion | 95% CI |
|---|---|---|---|---|---|---|
| Alexander | 1989 | 40 | 51 | 2.8% | 78.43 | [64.68; 88.71] |
| Benoist | 1993b | 33 | 42 | 2.7% | 78.57 | [63.19; 89.70] |
| Benoist (L) | 1993a | 36 | 41 | 2.3% | 87.80 | [73.80; 95.92] |
| Benoist (M) | 1993a | 38 | 43 | 2.3% | 88.37 | [74.92; 96.11] |
| Boccanera | 1990 | 54 | 60 | 2.5% | 90.00 | [79.49; 96.24] |
| Brown | 1989 | 35 | 51 | 2.9% | 68.63 | [54.11; 80.89] |
| Dabezies | 1985 | 272 | 382 | 3.4% | 71.20 | [66.38; 75.70] |
| Dabezies | 1987 | 44 | 62 | 3.0% | 70.97 | [58.05; 81.80] |
| Ejeskar | 1983 | 13 | 15 | 1.5% | 86.67 | [59.54; 98.34] |
| Gogan | 1991 | 24 | 30 | 2.4% | 80.00 | [61.43; 92.29] |
| Hill | 1986 | 261 | 335 | 3.3% | 77.91 | [73.08; 82.24] |
| Hofstra | 1989 | 15 | 15 | 0.6% | 100.00 | [78.20; 100.00] |
| Jaabay | 1985 | 221 | 263 | 3.3% | 84.03 | [79.03; 88.24] |
| Javid | 1983 | 40 | 55 | 2.9% | 72.73 | [59.04; 83.86] |
| Javid | 1985 | 85 | 105 | 3.1% | 80.95 | [72.13; 87.96] |
| Javid | 1992 | 90 | 104 | 2.9% | 86.54 | [78.45; 92.44] |
| Kato | 1993 | 27 | 28 | 1.1% | 96.43 | [81.65; 99.91] |
| LeBlanc | 1991 | 37 | 50 | 2.8% | 74.00 | [59.66; 85.37] |
| Leonardi | 1993 | 602 | 733 | 3.4% | 82.13 | [79.16; 84.84] |
| Leviseth | 1999 | 24 | 50 | 3.0% | 48.00 | [33.66; 62.58] |
| Lorenz | 1985 | 44 | 55 | 2.8% | 80.00 | [67.03; 89.57] |
| Louwaege | 1996 | 61 | 84 | 3.1% | 72.62 | [61.80; 81.79] |
| Maciunas | 1986 | 230 | 268 | 3.3% | 85.82 | [81.06; 89.77] |
| Maroon | 1976 | 39 | 48 | 2.7% | 81.25 | [67.37; 91.05] |
| McDermott | 1985 | 1226 | 1402 | 3.4% | 87.45 | [85.60; 89.14] |
| Nordby | 1972 | 74 | 100 | 3.1% | 74.00 | [64.27; 82.26] |
| Nordby | 1986 | 493 | 739 | 3.4% | 66.71 | [63.18; 70.10] |
| Parkinson | 1983 | 198 | 200 | 1.7% | 99.00 | [96.43; 99.88] |
| Ravichandran | 1980 | 20 | 26 | 2.4% | 76.92 | [56.35; 91.03] |
| Schwetschenau | 1976 | 15 | 31 | 2.7% | 48.39 | [30.15; 66.94] |
| Shields | 1987 | 50 | 126 | 3.2% | 39.68 | [31.08; 48.78] |
| Smith | 1967 | 68 | 75 | 2.6% | 90.67 | [81.71; 96.16] |
| Sutton | 1985a | 24 | 33 | 2.6% | 72.73 | [54.48; 86.70] |
| Sutton | 1985b | 147 | 189 | 3.3% | 77.78 | [71.17; 83.49] |
| Wardlaw | 2013b | 42 | 45 | 2.0% | 93.33 | [81.73; 98.60] |
| Wittenberg | 2001a | 36 | 50 | 2.9% | 72.00 | [57.51; 83.77] |
| Zeiger | 1987 | 27 | 45 | 2.9% | 60.00 | [44.33; 74.30] |
| **Total (95% CI)** | | **4785** | **6031** | **100.0%** | **78.82** | **[74.37; 82.67]** |

Heterogeneity: $Tau^2 = 0.4642$; $Chi^2 = 349.67$, df = 36 ($P < 0.01$); $I^2 = 90\%$

Events per 100 observations, IV, Random, 95% CI — axis: 40 50 60 70 80 90 100; Treatment success rate (chymopapain)

## Treatment success (Condoliase)

| Study | Year | Events | Total | Weight | Proportion | 95% CI |
|---|---|---|---|---|---|---|
| Banno | 2021 | 33 | 46 | 6.1% | 71.74 | [56.54; 84.01] |
| Banno | 2022 | 47 | 60 | 6.7% | 78.33 | [65.80; 87.93] |
| Banno | 2023 | 51 | 67 | 8.0% | 76.12 | [64.14; 85.69] |
| Chiba | 2018 | 65 | 82 | 8.9% | 79.27 | [68.89; 87.43] |
| Hirai | 2022 | 40 | 52 | 6.1% | 76.92 | [63.16; 87.47] |
| Inoue | 2021 | 65 | 84 | 9.7% | 77.38 | [66.95; 85.80] |
| Ishibashi | 2020 | 21 | 34 | 5.3% | 61.76 | [43.56; 77.83] |
| Kagami | 2023 | 121 | 157 | 18.0% | 77.07 | [69.70; 83.39] |
| Nakajima | 2020 | 32 | 42 | 5.0% | 76.19 | [60.55; 87.95] |
| Ohtonari | 2023 | 34 | 41 | 3.8% | 82.93 | [67.94; 92.85] |
| Okada | 2020 | 70 | 82 | 6.7% | 85.37 | [75.83; 92.20] |
| Oshita | 2022 | 55 | 71 | 8.1% | 77.46 | [66.00; 86.54] |
| Takeuchi | 2022 | 88 | 101 | 7.5% | 87.13 | [79.00; 92.96] |
| **Total (95% CI)** | | **722** | **919** | **.** | **78.11** | **[75.28; 80.71]** |

Heterogeneity: $Tau^2 = 0.0007$; $Chi^2 = 14.29$, df = 12 ($P = 0.28$); $I^2 = 16\%$

Events per 100 observations, IV, Fixed, 95% CI — axis: 50 60 70 80 90; Treatment success rate (condoliase)

## Treatment success (Collagenase)

| Study | Year | Events | Total | Weight | Proportion | 95% CI |
|---|---|---|---|---|---|---|
| Bromley | 1982 | 70 | 82 | 13.2% | 85.37 | [75.83; 92.20] |
| Bromley | 1983 | 40 | 46 | 11.4% | 86.96 | [73.74; 95.06] |
| Brown | 1985 | 34 | 54 | 13.6% | 62.96 | [48.74; 75.71] |
| Brown | 1989 | 10 | 15 | 9.9% | 66.67 | [38.38; 88.18] |
| Sussman | 1981 | 25 | 29 | 10.0% | 86.21 | [68.34; 96.11] |
| Wang | 2021 | 110 | 126 | 13.8% | 87.30 | [80.20; 92.56] |
| Wittenberg | 2001b | 26 | 50 | 13.6% | 52.00 | [37.42; 66.34] |
| Zhang | 2015 | 208 | 236 | 14.5% | 88.14 | [83.31; 91.97] |
| **Total (95% CI)** | | **523** | **638** | **100.0%** | **79.27** | **[68.63; 86.99]** |

Heterogeneity: $Tau^2 = 0.5188$; $Chi^2 = 49.15$, df = 7 ($P < 0.01$); $I^2 = 86\%$

Events per 100 observations, IV, Random, 95% CI — axis: 40 50 60 70 80 90; Treatment success rate (collagenase)

**Supplemental item 7.** Single-arm analysis of proceeding to surgery.

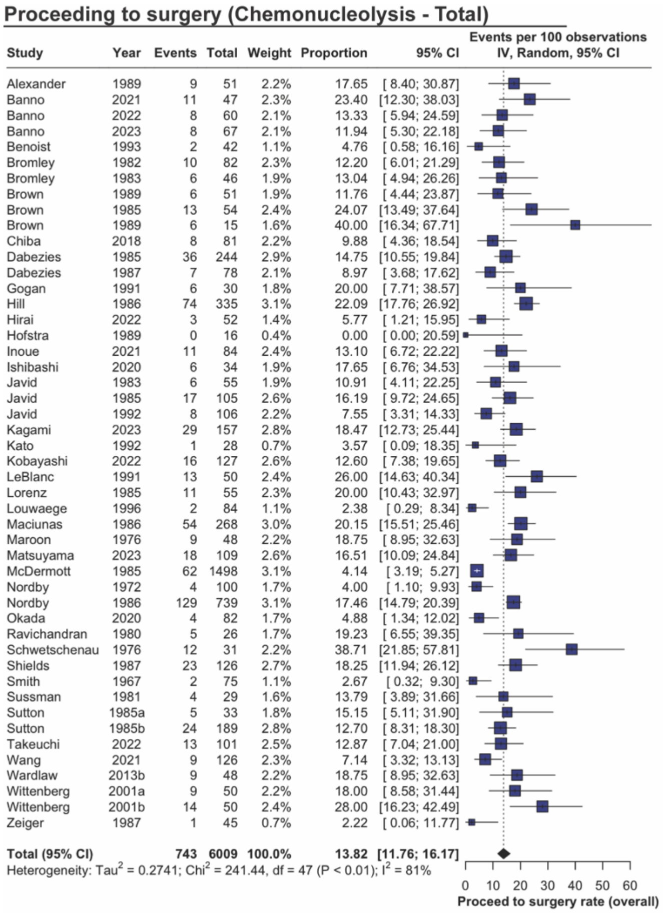


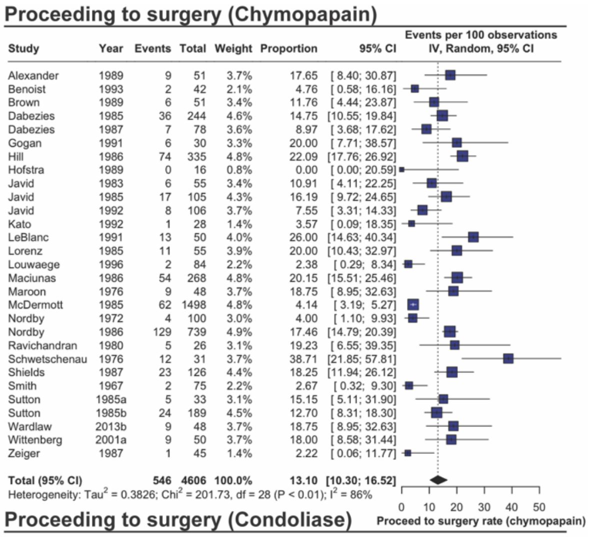


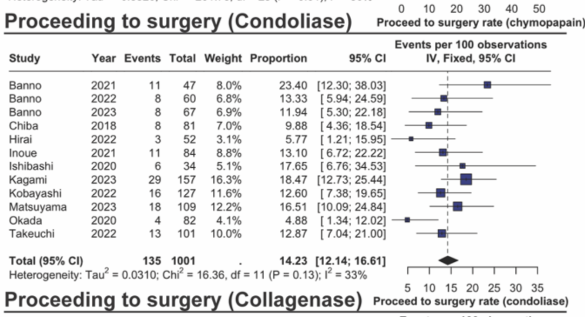


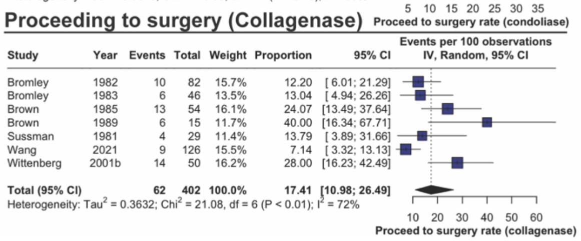

**Supplemental item 8.** Single-arm analysis of serious adverse events rates

## Serious Adverse Events (Chemonucleolysis - Total)

| Study | Year | Events | Total | Weight | Proportion | 95% CI |
|---|---|---|---|---|---|---|
| Alexander | 1989 | 2 | 51 | 2.6% | 3.92 | [ 0.48; 13.46] |
| Banno | 2021 | 0 | 47 | 1.3% | 0.00 | [ 0.00; 7.55] |
| Banno | 2022 | 0 | 60 | 1.3% | 0.00 | [ 0.00; 5.96] |
| Banno | 2023 | 0 | 67 | 1.3% | 0.00 | [ 0.00; 5.36] |
| Benoist | 1993b | 0 | 42 | 1.3% | 0.00 | [ 0.00; 8.41] |
| Benoist (L) | 1993a | 1 | 58 | 2.0% | 1.72 | [ 0.04; 9.24] |
| Benoist (M) | 1993a | 0 | 60 | 1.3% | 0.00 | [ 0.00; 5.96] |
| Boccanera | 1990 | 15 | 60 | 3.6% | 25.00 | [14.72; 37.86] |
| Bromley | 1982 | 0 | 82 | 1.3% | 0.00 | [ 0.00; 4.40] |
| Bromley | 1983 | 0 | 52 | 1.3% | 0.00 | [ 0.00; 6.85] |
| Brown | 1985 | 1 | 54 | 2.0% | 1.85 | [ 0.05; 9.89] |
| Chiba | 2018 | 4 | 82 | 3.1% | 4.88 | [ 1.34; 12.02] |
| Dabezies | 1987 | 1 | 78 | 2.0% | 1.28 | [ 0.03; 6.94] |
| Gogan | 1991 | 2 | 30 | 2.6% | 6.67 | [ 0.82; 22.07] |
| Hall | 1983 | 15 | 4282 | 3.6% | 0.35 | [ 0.20; 0.58] |
| Hill | 1986 | 2 | 335 | 2.6% | 0.60 | [ 0.07; 2.14] |
| Hirai | 2022 | 0 | 52 | 1.3% | 0.00 | [ 0.00; 6.85] |
| Hofstra | 1989 | 0 | 16 | 1.3% | 0.00 | [ 0.00; 20.59] |
| Inoue | 2021 | 2 | 84 | 2.6% | 2.38 | [ 0.29; 8.34] |
| Javid | 1983 | 0 | 55 | 1.3% | 0.00 | [ 0.00; 6.49] |
| Javid | 1992 | 0 | 106 | 1.3% | 0.00 | [ 0.00; 3.42] |
| Kobayashi | 2022 | 0 | 127 | 1.3% | 0.00 | [ 0.00; 2.86] |
| Kobayashi | 2023 | 0 | 26 | 1.3% | 0.00 | [ 0.00; 13.23] |
| LeBlanc | 1991 | 1 | 50 | 2.0% | 2.00 | [ 0.05; 10.65] |
| Leonardi | 1993 | 1 | 733 | 2.0% | 0.14 | [ 0.00; 0.76] |
| Lorenz | 1985 | 3 | 55 | 2.9% | 5.45 | [ 1.14; 15.12] |
| Louwaege | 1996 | 1 | 84 | 2.0% | 1.19 | [ 0.03; 6.46] |
| Maciunas | 1986 | 0 | 268 | 1.3% | 0.00 | [ 0.00; 1.37] |
| Maroon | 1976 | 4 | 48 | 3.1% | 8.33 | [ 2.32; 19.98] |
| Matsuyama (H) | 2018 | 0 | 49 | 1.3% | 0.00 | [ 0.00; 7.25] |
| Matsuyama (L) | 2018 | 0 | 49 | 1.3% | 0.00 | [ 0.00; 7.25] |
| Matsuyama (M) | 2018 | 0 | 49 | 1.3% | 0.00 | [ 0.00; 7.25] |
| McDermott | 1985 | 73 | 1498 | 3.8% | 4.87 | [ 3.84; 6.09] |
| Nakajima | 2020 | 0 | 42 | 1.3% | 0.00 | [ 0.00; 8.41] |
| Nordby | 1972 | 0 | 100 | 1.3% | 0.00 | [ 0.00; 3.62] |
| Nordby | 1986 | 3 | 748 | 2.9% | 0.40 | [ 0.08; 1.17] |
| Okada | 2020 | 0 | 82 | 1.3% | 0.00 | [ 0.00; 4.40] |
| Parkinson | 1983 | 0 | 33 | 1.3% | 0.00 | [ 0.00; 10.58] |
| Ravichandran | 1980 | 0 | 26 | 1.3% | 0.00 | [ 0.00; 13.23] |
| Schwetschenau | 1976 | 0 | 31 | 1.3% | 0.00 | [ 0.00; 11.22] |
| Shields | 1987 | 1 | 150 | 2.0% | 0.67 | [ 0.02; 3.66] |
| Smith | 1967 | 3 | 75 | 2.9% | 4.00 | [ 0.83; 11.25] |
| Sussman | 1981 | 0 | 29 | 1.3% | 0.00 | [ 0.00; 11.94] |
| Sutton | 1985a | 3 | 33 | 2.9% | 9.09 | [ 1.92; 24.33] |
| Sutton | 1985b | 17 | 189 | 3.6% | 8.99 | [ 5.33; 14.01] |
| Takeuchi | 2022 | 0 | 101 | 1.3% | 0.00 | [ 0.00; 3.59] |
| Wang | 2021 | 1 | 126 | 2.0% | 0.79 | [ 0.02; 4.34] |
| Wardlaw | 2013 | 1 | 48 | 2.0% | 2.08 | [ 0.05; 11.07] |
| Wittenberg | 2001a | 1 | 50 | 2.0% | 2.00 | [ 0.05; 10.65] |
| Wittenberg | 2001b | 0 | 50 | 1.3% | 0.00 | [ 0.00; 7.11] |
| Zeiger | 1987 | 1 | 45 | 2.0% | 2.22 | [ 0.06; 11.77] |
| Zhang | 2015 | 0 | 236 | 1.3% | 0.00 | [ 0.00; 1.55] |
| **Total (95% CI)** | | **159** | **10983** | . | **3.46** | **[ 2.98; 4.01]** |

Heterogeneity: $Tau^2 = 1.0589$; $Chi^2 = 242.78$, df = 51 ($P < 0.01$); $I^2 = 79\%$

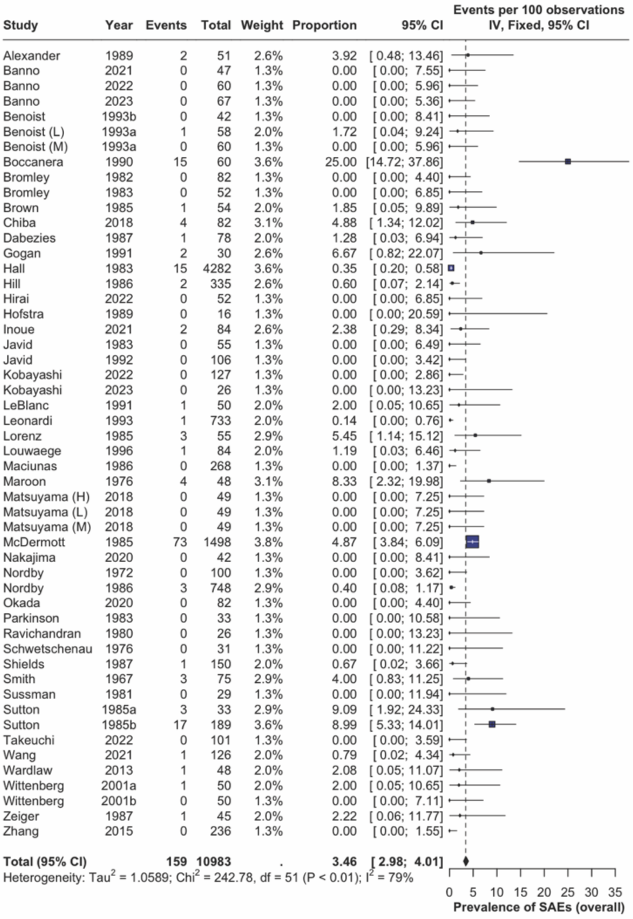


## Serious Adverse Events (Chymopapain)

| Study | Year | Events | Total | Weight | Proportion | 95% CI |
|---|---|---|---|---|---|---|
| Alexander | 1989 | 2 | 51 | 3.8% | 3.92 | [ 0.48; 13.46] |
| Benoist | 1993b | 0 | 42 | 2.1% | 0.00 | [ 0.00; 8.41] |
| Benoist (L) | 1993a | 1 | 58 | 3.0% | 1.72 | [ 0.04; 9.24] |
| Benoist (M) | 1993a | 0 | 60 | 2.1% | 0.00 | [ 0.00; 5.96] |
| Boccanera | 1990 | 15 | 60 | 4.9% | 25.00 | [14.72; 37.86] |
| Dabezies | 1987 | 1 | 78 | 3.0% | 1.28 | [ 0.03; 6.94] |
| Gogan | 1991 | 2 | 30 | 3.7% | 6.67 | [ 0.82; 22.07] |
| Hall | 1983 | 15 | 4282 | 5.0% | 0.35 | [ 0.20; 0.58] |
| Hill | 1986 | 2 | 335 | 3.8% | 0.60 | [ 0.07; 2.14] |
| Hofstra | 1989 | 0 | 16 | 2.1% | 0.00 | [ 0.00; 20.59] |
| Javid | 1983 | 0 | 55 | 2.1% | 0.00 | [ 0.00; 6.49] |
| Javid | 1992 | 0 | 106 | 2.1% | 0.00 | [ 0.00; 3.42] |
| LeBlanc | 1991 | 1 | 50 | 3.0% | 2.00 | [ 0.05; 10.65] |
| Leonardi | 1993 | 1 | 733 | 3.0% | 0.14 | [ 0.00; 0.76] |
| Lorenz | 1985 | 3 | 55 | 4.1% | 5.45 | [ 1.14; 15.12] |
| Louwaege | 1996 | 1 | 84 | 3.0% | 1.19 | [ 0.03; 6.46] |
| Maciunas | 1986 | 0 | 268 | 2.1% | 0.00 | [ 0.00; 1.37] |
| Maroon | 1976 | 4 | 48 | 4.3% | 8.33 | [ 2.32; 19.98] |
| McDermott | 1985 | 73 | 1498 | 5.1% | 4.87 | [ 3.84; 6.09] |
| Nordby | 1972 | 0 | 100 | 2.1% | 0.00 | [ 0.00; 3.62] |
| Nordby | 1986 | 3 | 748 | 4.2% | 0.40 | [ 0.08; 1.17] |
| Parkinson | 1983 | 0 | 33 | 2.1% | 0.00 | [ 0.00; 10.58] |
| Ravichandran | 1980 | 0 | 26 | 2.1% | 0.00 | [ 0.00; 13.23] |
| Schwetschenau | 1976 | 0 | 31 | 2.1% | 0.00 | [ 0.00; 11.22] |
| Shields | 1987 | 1 | 150 | 3.0% | 0.67 | [ 0.02; 3.66] |
| Smith | 1967 | 3 | 75 | 4.1% | 4.00 | [ 0.83; 11.25] |
| Sutton | 1985a | 3 | 33 | 4.1% | 9.09 | [ 1.92; 24.33] |
| Sutton | 1985b | 17 | 189 | 5.0% | 8.99 | [ 5.33; 14.01] |
| Wardlaw | 2013b | 1 | 48 | 3.0% | 2.08 | [ 0.05; 11.07] |
| Wittenberg | 2001 | 1 | 50 | 3.0% | 2.00 | [ 0.05; 10.65] |
| Zeiger | 1987 | 1 | 45 | 3.0% | 2.22 | [ 0.06; 11.77] |
| **Total (95% CI)** | | **151** | **9437** | **100.0%** | **2.06** | **[ 1.23; 3.42]** |

Heterogeneity: $Tau^2 = 1.3650$; $Chi^2 = 217.43$, df = 30 ($P < 0.01$); $I^2 = 86\%$

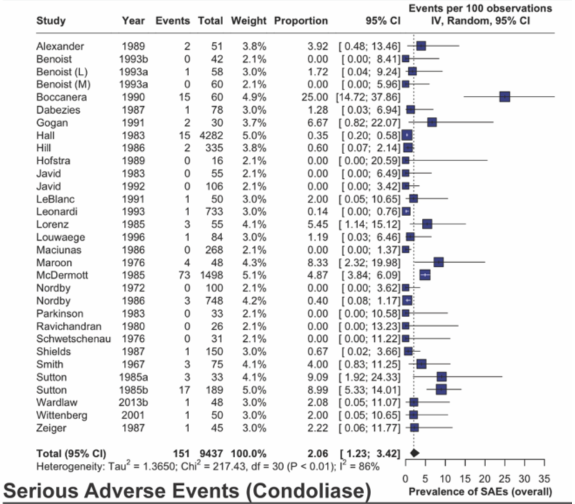


## Serious Adverse Events (Condoliase)

| Study | Year | Events | Total | Weight | Proportion | 95% CI |
|---|---|---|---|---|---|---|
| Banno | 2021 | 0 | 47 | 5.4% | 0.00 | [0.00; 7.58] |
| Banno | 2022 | 0 | 60 | 6.7% | 0.00 | [0.00; 5.81] |
| Banno | 2023 | 0 | 67 | 7.4% | 0.00 | [0.00; 5.13] |
| Chiba | 2018 | 4 | 82 | 8.8% | 4.88 | [1.34; 12.02] |
| Hirai | 2022 | 0 | 52 | 5.9% | 0.00 | [0.06; 7.13] |
| Inoue | 2021 | 2 | 84 | 9.0% | 2.38 | [0.04; 7.87] |
| Kobayashi | 2022 | 0 | 127 | 12.6% | 0.00 | [0.00; 2.69] |
| Kobayashi | 2023 | 0 | 26 | 3.2% | 0.00 | [0.19; 13.80] |
| Matsuyama (H) | 2018 | 0 | 49 | 5.6% | 0.00 | [0.13; 7.67] |
| Matsuyama (L) | 2018 | 0 | 49 | 5.6% | 0.00 | [0.19; 7.75] |
| Matsuyama (M) | 2018 | 0 | 49 | 5.6% | 0.00 | [0.00; 7.30] |
| Nakajima | 2020 | 0 | 42 | 4.9% | 0.00 | [0.05; 8.68] |
| Okada | 2020 | 0 | 82 | 8.8% | 0.00 | [0.00; 4.28] |
| Takeuchi | 2022 | 0 | 101 | 10.5% | 0.00 | [0.00; 2.47] |
| **Total (95% CI)** | | **6** | **917** | . | **0.14** | **[0.00; 0.69]** |

Heterogeneity: $Tau^2 = 0.0005$; $Chi^2 = 12.05$, df = 13 ($P = 0.52$); $I^2 = 0\%$

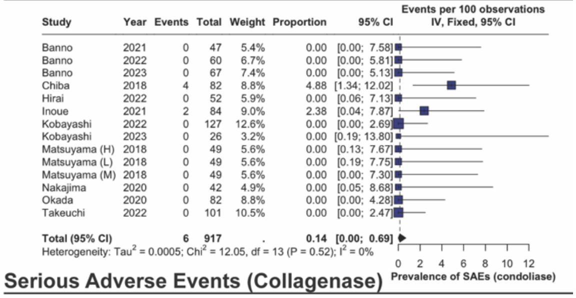


## Serious Adverse Events (Collagenase)

| Study | Year | Events | Total | Weight | Proportion | 95% CI |
|---|---|---|---|---|---|---|
| Bromley | 1982 | 0 | 82 | 13.2% | 0.00 | [0.00; 3.52] |
| Bromley | 1983 | 0 | 52 | 8.5% | 0.00 | [0.06; 7.13] |
| Brown | 1985 | 1 | 54 | 8.8% | 1.85 | [0.04; 9.86] |
| Sussman | 1981 | 0 | 29 | 4.8% | 0.00 | [0.18; 12.50] |
| Wang | 2021 | 1 | 126 | 20.1% | 0.79 | [0.10; 4.50] |
| Wittenberg | 2001b | 0 | 50 | 8.2% | 0.00 | [0.18; 7.60] |
| Zhang | 2015 | 0 | 236 | 36.4% | 0.00 | [0.00; 0.92] |
| **Total (95% CI)** | | **2** | **629** | . | **0.01** | **[0.00; 0.52]** |

Heterogeneity: $Tau^2 < 0.0001$; $Chi^2 = 4.07$, df = 6 ($P = 0.67$); $I^2 = 0\%$

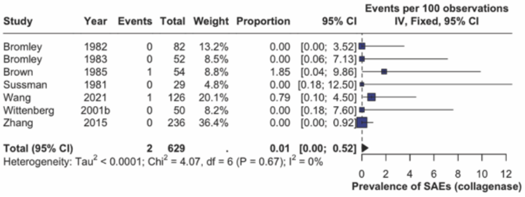

**Supplemental item 9.** Meta-analysis forest plots for serious adverse events in chemonucleolysis treatment versus placebo or discectomy-treated cohorts.

## Odds ratio assessment for "serious adverse events"

| | Chemonucleolysis | | Placebo | | | Odds Ratio |
|---|---|---|---|---|---|---|
| **Study or Subgroup** | **Events** | **Total** | **Events** | **Total** | **Weight** | **M–H, Fixed, 95% CI** |
| Chiba 2018 | 4 | 82 | 6 | 82 | 36.1% | 0.65 [0.18, 2.39] |
| Dabezies 1985 | 1 | 78 | 0 | 81 | 3.0% | 3.15 [0.13, 78.62] |
| Gogan 1991 | 2 | 30 | 6 | 30 | 35.4% | 0.29 [0.05, 1.55] |
| Javid 1983 | 0 | 55 | 1 | 53 | 9.6% | 0.32 [0.01, 7.91] |
| Matsuyama 2018 | 0 | 49 | 2 | 47 | 16.0% | 0.18 [0.01, 3.93] |
| Schwetschenau 1976 | 0 | 31 | 0 | 35 | | Not estimable |
| **Total (95% CI)** | | **325** | | **328** | **100.0%** | **0.49 [0.21, 1.15]** |
| Total events | 7 | | 15 | | | |

Heterogeneity: Chi$^2$ = 2.32, df = 4 (P = 0.68); I$^2$ = 0%
Test for overall effect: Z = 1.64 (P = 0.10)

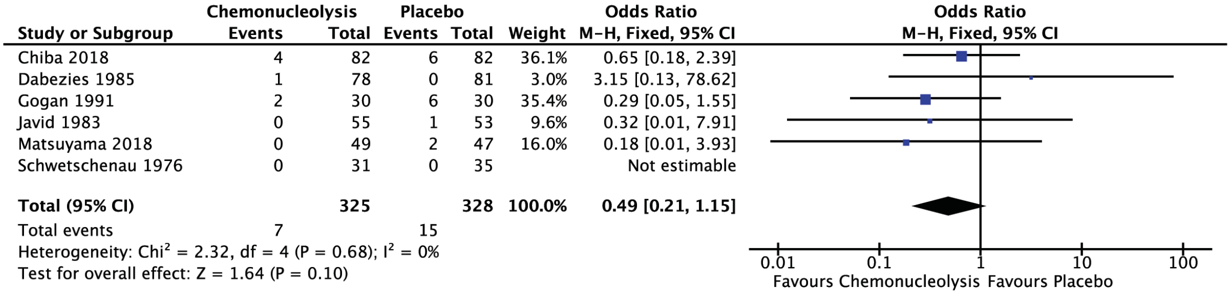


| | Chemonucleolysis | | Surgery | | | Odds Ratio |
|---|---|---|---|---|---|---|
| **Study or Subgroup** | **Events** | **Total** | **Events** | **Total** | **Weight** | **M–H, Fixed, 95% CI** |
| Alexander 1989 | 2 | 51 | 5 | 49 | 48.1% | 0.36 [0.07, 1.95] |
| Javid 1992 | 0 | 106 | 0 | 72 | | Not estimable |
| Nordby 1972 | 0 | 100 | 3 | 91 | 35.8% | 0.13 [0.01, 2.47] |
| Wardlaw 2013b | 1 | 48 | 1 | 52 | 9.2% | 1.09 [0.07, 17.84] |
| Zeiger 1987 | 1 | 45 | 1 | 81 | 6.9% | 1.82 [0.11, 29.78] |
| **Total (95% CI)** | | **350** | | **345** | **100.0%** | **0.44 [0.15, 1.34]** |
| Total events | 4 | | 10 | | | |

Heterogeneity: Chi$^2$ = 2.12, df = 3 (P = 0.55); I$^2$ = 0%
Test for overall effect: Z = 1.44 (P = 0.15)

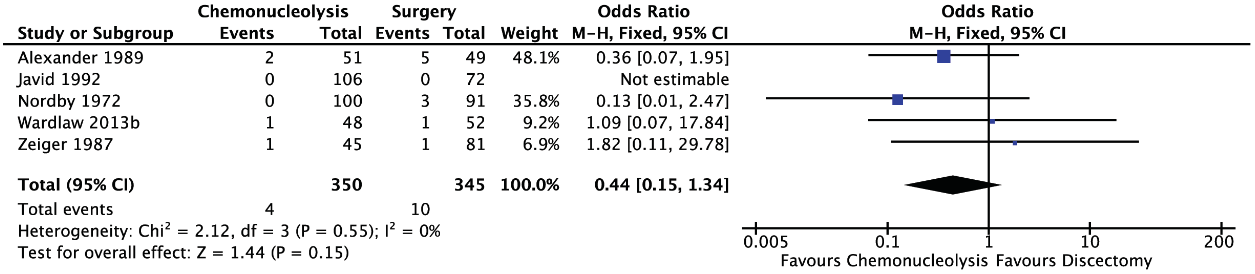

**Supplemental item 10.** Single-arm analysis of allergic reactions

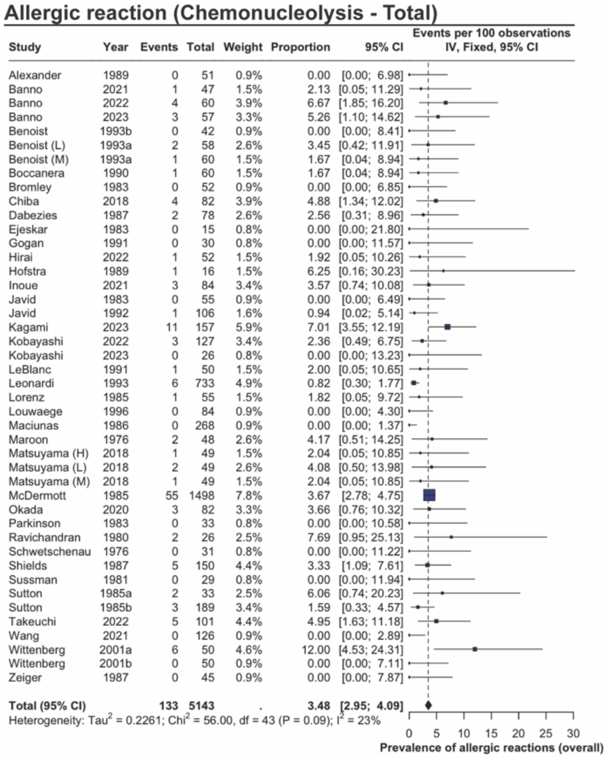


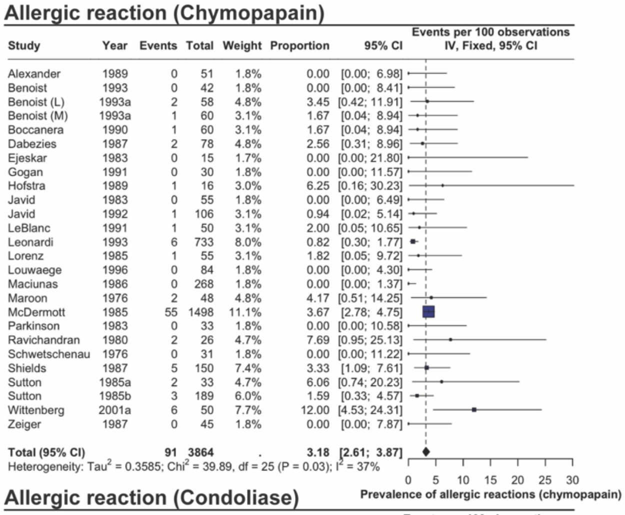


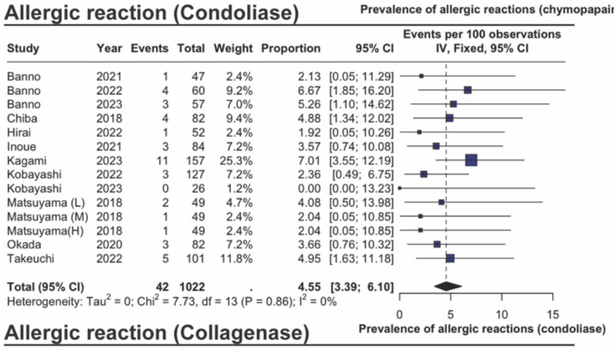


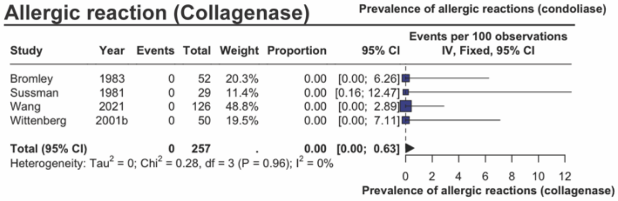

**Supplemental item 11.** Single-arm analysis of anaphylactic shock.

## Anaphylaxis (Chemonucleolysis - Total)

| Study | Year | Events | Total | Weight | Proportion | 95% CI |
|---|---|---|---|---|---|---|
| Alexander | 1989 | 0 | 51 | 0.8% | 0.00 | [0.05; 7.23] |
| Banno | 2021 | 0 | 47 | 0.7% | 0.00 | [0.00; 7.13] |
| Banno | 2022 | 0 | 60 | 0.9% | 0.00 | [0.00; 5.80] |
| Banno | 2023 | 0 | 67 | 1.0% | 0.00 | [0.00; 4.44] |
| Benoist | 1993b | 0 | 42 | 0.7% | 0.00 | [0.03; 8.62] |
| Benoist (L) | 1993a | 1 | 58 | 0.9% | 1.72 | [0.57; 9.90] |
| Benoist (M) | 1993a | 0 | 60 | 0.9% | 0.00 | [0.00; 4.14] |
| Boccanera | 1990 | 0 | 60 | 0.9% | 0.00 | [0.00; 5.43] |
| Bromley | 1982 | 0 | 82 | 1.2% | 0.00 | [0.00; 3.67] |
| Bromley | 1983 | 0 | 52 | 0.8% | 0.00 | [0.25; 7.41] |
| Brown | 1985 | 0 | 54 | 0.8% | 0.00 | [0.42; 7.34] |
| Chiba | 2018 | 0 | 82 | 1.2% | 0.00 | [0.00; 4.15] |
| Dabezies | 1987 | 1 | 78 | 1.2% | 1.28 | [0.37; 7.41] |
| Ejeskar | 1983 | 0 | 15 | 0.2% | 0.00 | [1.41; 23.39] |
| Gogan | 1991 | 0 | 30 | 0.5% | 0.00 | [0.67; 12.66] |
| Hall | 1983 | 15 | 4282 | 17.0% | 0.35 | [0.00; 0.13] |
| Hill | 1986 | 1 | 335 | 4.3% | 0.30 | [0.00; 1.49] |
| Hirai | 2022 | 0 | 52 | 0.8% | 0.00 | [0.00; 6.73] |
| Hofstra | 1989 | 0 | 16 | 0.3% | 0.00 | [0.00; 20.59] |
| Inoue | 2021 | 0 | 84 | 1.3% | 0.00 | [0.00; 3.98] |
| Javid | 1983 | 0 | 55 | 0.8% | 0.00 | [0.00; 6.55] |
| Javid | 1992 | 0 | 106 | 1.6% | 0.00 | [0.00; 2.47] |
| Kagami | 2023 | 0 | 157 | 2.3% | 0.00 | [0.00; 1.76] |
| Kobayashi | 2022 | 0 | 127 | 1.9% | 0.00 | [0.00; 2.79] |
| Kobayashi | 2023 | 0 | 26 | 0.4% | 0.00 | [0.28; 13.94] |
| LeBlanc | 1991 | 0 | 50 | 0.8% | 0.00 | [0.01; 7.24] |
| Leonardi | 1993 | 0 | 733 | 7.7% | 0.00 | [0.00; 0.50] |
| Lorenz | 1985 | 1 | 55 | 0.8% | 1.82 | [0.00; 9.51] |
| Louwaege | 1996 | 0 | 84 | 1.3% | 0.00 | [0.00; 4.30] |
| Maciunas | 1986 | 0 | 268 | 3.6% | 0.00 | [0.00; 0.73] |
| Maroon | 1976 | 0 | 48 | 0.7% | 0.00 | [0.00; 7.40] |
| Matsuyama (H) | 2018 | 0 | 49 | 0.8% | 0.00 | [0.00; 7.25] |
| Matsuyama (L) | 2018 | 0 | 49 | 0.8% | 0.00 | [0.00; 7.25] |
| Matsuyama (M) | 2018 | 0 | 49 | 0.8% | 0.00 | [0.00; 7.25] |
| McDermott | 1985 | 14 | 1498 | 11.6% | 0.93 | [0.11; 1.06] |
| Nakajima | 2020 | 0 | 42 | 0.7% | 0.00 | [0.00; 8.41] |
| Nordby | 1986 | 2 | 748 | 7.8% | 0.27 | [0.00; 0.52] |
| Okada | 2020 | 0 | 82 | 1.2% | 0.00 | [0.00; 4.41] |
| Parkinson | 1983 | 0 | 33 | 0.5% | 0.00 | [0.07; 10.90] |
| Ravichandran | 1980 | 0 | 26 | 0.4% | 0.00 | [0.51; 14.19] |
| Schwetschenau | 1976 | 0 | 31 | 0.5% | 0.00 | [0.45; 12.09] |
| Shields | 1987 | 0 | 150 | 2.2% | 0.00 | [0.00; 1.91] |
| Smith | 1967 | 1 | 75 | 1.1% | 1.33 | [0.00; 7.07] |
| Sussman | 1981 | 0 | 29 | 0.5% | 0.00 | [0.27; 12.63] |
| Sutton | 1985a | 1 | 33 | 0.5% | 3.03 | [0.73; 16.53] |
| Sutton | 1985b | 1 | 189 | 2.7% | 0.53 | [0.00; 1.63] |
| Takeuchi | 2022 | 0 | 101 | 1.5% | 0.00 | [0.00; 2.61] |
| Wang | 2021 | 0 | 126 | 1.8% | 0.00 | [0.00; 2.70] |
| Wardlaw | 2013b | 0 | 48 | 0.7% | 0.00 | [0.00; 7.46] |
| Wittenberg | 2001a | 0 | 50 | 0.8% | 0.00 | [0.00; 7.17] |
| Wittenberg | 2001b | 0 | 50 | 0.8% | 0.00 | [0.31; 7.75] |
| Zeiger | 1987 | 0 | 45 | 0.7% | 0.00 | [0.23; 8.43] |
| Zhang | 2015 | 0 | 236 | 3.2% | 0.00 | [0.00; 0.92] |
| **Total (95% CI)** | | **38** | **11055** | . | **0.00** | **[0.00; 0.02]** |

Heterogeneity: $Tau^2 = 0.0002$; $Chi^2 = 29.11$, df = 52 (P = 1.00); $I^2 = 0\%$

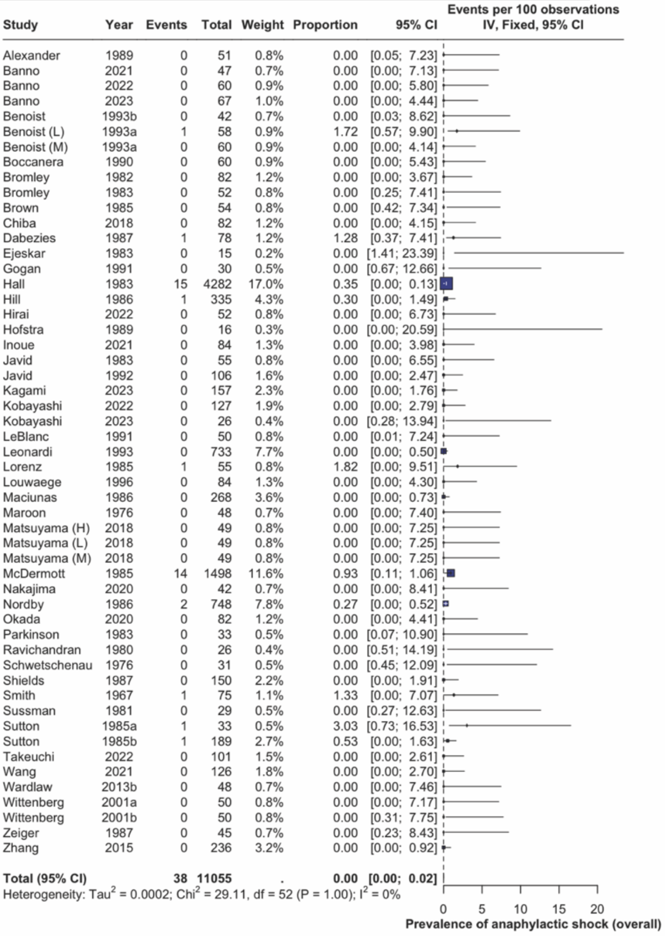


## Anaphylaxis (Chymopapain)

| Study | Year | Events | Total | Weight | Proportion | 95% CI |
|---|---|---|---|---|---|---|
| Alexander | 1989 | 0 | 51 | 1.3% | 0.00 | [0.05; 7.23] |
| Benoist | 1993b | 0 | 42 | 1.1% | 0.00 | [0.00; 8.09] |
| Benoist (L) | 1993a | 1 | 58 | 1.5% | 1.72 | [0.01; 9.10] |
| Benoist (M) | 1993a | 0 | 60 | 1.5% | 0.00 | [0.00; 5.12] |
| Boccanera | 1990 | 0 | 60 | 1.5% | 0.00 | [0.00; 5.90] |
| Dabezies | 1987 | 1 | 78 | 1.9% | 1.28 | [0.42; 7.46] |
| Ejeskar | 1983 | 0 | 15 | 0.4% | 0.00 | [0.00; 21.80] |
| Gogan | 1991 | 0 | 30 | 0.8% | 0.00 | [0.01; 11.68] |
| Hall | 1983 | 15 | 4282 | 17.6% | 0.35 | [0.00; 0.00] |
| Hill | 1986 | 1 | 335 | 6.3% | 0.30 | [0.00; 1.55] |
| Hofstra | 1989 | 0 | 16 | 0.4% | 0.00 | [1.46; 22.23] |
| Javid | 1983 | 0 | 55 | 1.4% | 0.00 | [0.00; 6.49] |
| Javid | 1992 | 0 | 106 | 2.5% | 0.00 | [0.17; 3.78] |
| LeBlanc | 1991 | 0 | 50 | 1.3% | 0.00 | [0.33; 7.77] |
| Leonardi | 1993 | 0 | 733 | 10.1% | 0.00 | [0.00; 0.43] |
| Lorenz | 1985 | 1 | 55 | 1.4% | 1.82 | [0.15; 9.92] |
| Louwaege | 1996 | 0 | 84 | 2.1% | 0.00 | [0.06; 4.52] |
| Maciunas | 1986 | 0 | 268 | 5.4% | 0.00 | [0.00; 0.66] |
| Maroon | 1976 | 0 | 48 | 1.2% | 0.00 | [0.00; 5.88] |
| McDermott | 1985 | 14 | 1498 | 13.7% | 0.93 | [0.01; 0.79] |
| Nordby | 1986 | 2 | 748 | 10.2% | 0.27 | [0.00; 0.38] |
| Parkinson | 1983 | 0 | 33 | 0.9% | 0.00 | [0.00; 10.47] |
| Ravichandran | 1980 | 0 | 26 | 0.7% | 0.00 | [0.21; 13.85] |
| Schwetschenau | 1976 | 0 | 31 | 0.8% | 0.00 | [0.39; 12.04] |
| Shields | 1987 | 0 | 150 | 3.4% | 0.00 | [0.00; 1.98] |
| Smith | 1967 | 1 | 75 | 1.9% | 1.33 | [0.00; 7.07] |
| Sutton | 1985a | 1 | 33 | 0.9% | 3.03 | [0.95; 16.70] |
| Sutton | 1985b | 1 | 189 | 4.1% | 0.53 | [0.00; 2.15] |
| Wardlaw | 2013b | 0 | 48 | 1.2% | 0.00 | [0.09; 7.75] |
| Wittenberg | 2001a | 0 | 50 | 1.3% | 0.00 | [0.00; 7.11] |
| Zeiger | 1987 | 0 | 45 | 1.2% | 0.00 | [0.00; 7.92] |
| **Total (95% CI)** | | **38** | **9352** | . | **0.00** | **[0.00; 0.02]** |

Heterogeneity: $Tau^2 = 0.0003$; $Chi^2 = 26.72$, df = 30 (P = 0.64); $I^2 = 0\%$

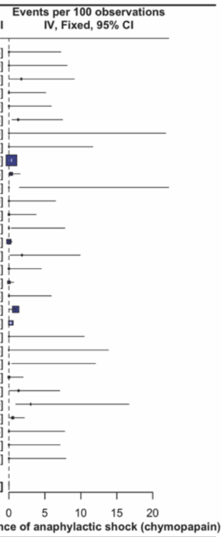


## Anaphylaxis (Condoliase)

| Study | Year | Events | Total | Weight | Proportion | 95% CI |
|---|---|---|---|---|---|---|
| Banno | 2021 | 0 | 47 | 4.4% | 0.00 | [0.00; 7.58] |
| Banno | 2022 | 0 | 60 | 5.6% | 0.00 | [0.00; 5.81] |
| Banno | 2023 | 0 | 67 | 6.2% | 0.00 | [0.00; 5.13] |
| Chiba | 2018 | 0 | 82 | 7.6% | 0.00 | [0.00; 4.40] |
| Hirai | 2022 | 0 | 52 | 4.9% | 0.00 | [0.00; 6.67] |
| Inoue | 2021 | 0 | 84 | 7.8% | 0.00 | [0.00; 4.28] |
| Kagami | 2023 | 0 | 157 | 14.6% | 0.00 | [0.00; 2.08] |
| Kobayashi | 2022 | 0 | 127 | 11.8% | 0.00 | [0.00; 2.31] |
| Kobayashi | 2023 | 0 | 26 | 2.5% | 0.00 | [0.51; 14.19] |
| Matsuyama (H) | 2018 | 0 | 49 | 4.6% | 0.00 | [0.19; 7.75] |
| Matsuyama (L) | 2018 | 0 | 49 | 4.6% | 0.00 | [0.00; 7.30] |
| Matsuyama (M) | 2018 | 0 | 49 | 4.6% | 0.00 | [0.02; 7.40] |
| Nakajima | 2020 | 0 | 42 | 3.9% | 0.00 | [0.08; 8.75] |
| Okada | 2020 | 0 | 82 | 7.6% | 0.00 | [0.07; 4.65] |
| Takeuchi | 2022 | 0 | 101 | 9.4% | 0.00 | [0.00; 2.47] |
| **Total (95% CI)** | | **0** | **1074** | . | **0.00** | **[0.00; 0.15]** |

Heterogeneity: $Tau^2 = 0$; $Chi^2 = 0.74$, df = 14 (P = 1.00); $I^2 = 0\%$

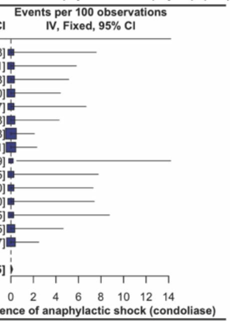


## Anaphylaxis (Collagenase)

| Study | Year | Events | Total | Weight | Proportion | 95% CI |
|---|---|---|---|---|---|---|
| Bromley | 1982 | 0 | 82 | 13.0% | 0.00 | [0.00; 3.52] |
| Bromley | 1983 | 0 | 52 | 8.3% | 0.00 | [0.06; 7.13] |
| Brown | 1985 | 0 | 54 | 8.6% | 0.00 | [0.00; 6.57] |
| Sussman | 1981 | 0 | 29 | 4.7% | 0.00 | [0.18; 12.50] |
| Wang | 2021 | 0 | 126 | 20.0% | 0.00 | [0.04; 3.05] |
| Wittenberg | 2001b | 0 | 50 | 8.0% | 0.00 | [0.18; 7.60] |
| Zhang | 2015 | 0 | 236 | 37.4% | 0.00 | [0.00; 0.92] |
| **Total (95% CI)** | | **0** | **629** | . | **0.00** | **[0.00; 0.18]** |

Heterogeneity: $Tau^2 = 0$; $Chi^2 = 0.75$, df = 6 (P = 0.99); $I^2 = 0\%$

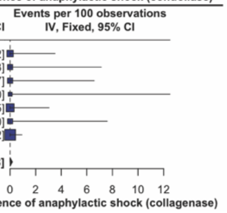

Supplement: Supplementary file 1 — Supplementary Information. [file 41598_2024_62792_MOESM1_ESM.pdf]
